# PorA, a conserved C-terminal domain-containing protein, impacts the PorXY-SigP signaling of the type IX secretion system

**DOI:** 10.1038/s41598-020-77987-y

**Published:** 2020-12-03

**Authors:** Hideharu Yukitake, Mikio Shoji, Keiko Sato, Yusuke Handa, Mariko Naito, Katsumi Imada, Koji Nakayama

**Affiliations:** 1 Department of Microbiology and Oral Infection, Graduate School of Biomedical Sciences, Nagasaki University, Nagasaki, Nagasaki 852-8588 Japan; 2 Department of Macromolecular Science, Graduate School of Science, Osaka University, Toyonaka, Osaka 560-0043 Japan

**Keywords:** Microbiology, Molecular biology

## Abstract

*Porphyromonas gingivalis*, a periodontal pathogen, translocates many virulence factors including the cysteine proteases referred to as gingipains to the cell surface via the type IX secretion system (T9SS). Expression of the T9SS component proteins is regulated by the tandem signaling of the PorXY two-component system and the ECF sigma factor SigP. However, the details of this regulatory pathway are still unknown. We found that one of the T9SS conserved C-terminal domain-containing proteins, PGN_0123, which we have designated PorA, is involved in regulating expression of genes encoding T9SS structural proteins and that PorA can be translocated onto the cell surface without the T9SS translocation machinery. X-ray crystallography revealed that PorA has a domain similar to the mannose-binding domain of *Escherichia coli* FimH, the tip protein of Type 1 pilus. Mutations in the cytoplasmic domain of the sensor kinase PorY conferred phenotypic recovery on the *ΔporA* mutant. The SigP sigma factor, which is activated by the PorXY two-component system, markedly decreased in the *ΔporA* mutant. These results strongly support a potential role for PorA in relaying a signal from the cell surface to the PorXY-SigP signaling pathway.

## Introduction

Chronic periodontitis is caused by bacterial infection and the subsequent immune response. A gram-negative anaerobe, *Porphyromonas gingivalis*, is a keystone pathogen of chronic periodontitis^1^. Gingipains are cysteine proteases located on the cell surface, which are central to virulence and hemagglutinating activity^2,3^. *P. gingivalis* has two types of gingipains; arginine-specific proteases, RgpA and RgpB, and lysine-specific protease, Kgp. Gingipains are translocated to the cell surface or in milieu by using the Type IX secretion system (T9SS)^4,5^. Approximately 30 proteins, including gingipains, Hbp35, TapA, PepK, PPAD, Mfa5, PorU, and PorZ, are secreted via the T9SS^6–8^ and are called T9SS cargo proteins. However, most of the T9SS cargo proteins have not been characterized.

The T9SS cargo proteins share a conserved C-terminal domain (CTD) composed of about 80 amino acid residues as a secretion signal for the T9SS. Sequence analysis of CTDs have revealed that T9SS CTDs belong to either protein domain family TIGR04183 (type A CTDs) or TIGR04131 (type B CTDs)^9^. Most of the T9SS cargo proteins have type A CTDs, but *Flavobacterium johnsoniae* has many T9SS cargo proteins with type B CTDs^10^. *P. gingivalis* has only one T9SS cargo protein with type B CTD (PGN_1317) and the others with type A CTDs. *P. gingivalis* T9SS cargo proteins can be classified into three groups according to their anchoring modes on the cell surface^2,6,8,11–21^. Group I proteins, which include RgpB, Hbp35, TapA, PepK, and PPAD, are covalently attached to anionic polysaccharide-containing lipopolysaccharides (A-LPS) after removal of their CTDs by the PorU sortase^22^. The attachment to A-LPS yields diffuse protein bands of the group I proteins in SDS-PAGE. Group II proteins are associated with other proteins, not A-LPS on the cell surface after removal of their CTDs by PorU, which include Mfa5. Mature/processed RgpA and Kgp may be categorized in this group; however, the proteins are generated by the proteolytic processing of RgpA and Kgp precursors. Group III proteins possess their CTDs on cell surface and are not associated with A-LPS. PorU and PorZ, which are T9SS component proteins, belong to group III^11,16^. PorU cleaves CTD of groups I and II cargo proteins, but no common sequence has been found at the cleavage sites^23^. It is still unknown how PorU recognizes the cleavage site.

*Porphyromonas gingivalis* colonies grown on blood agar plates are black-pigmented, and many genes responsible for this unique characteristic of the bacterium have been identified. The pigmentation is caused by release of heme from hemoglobin by Kgp^24,25^ and subsequent accumulation of a heme derivative, μ-oxo heme dimer, on the cell surface^26^. Transposon mutagenesis revealed that *porR* and *porT* genes are involved in the pigmentation^4,27^. PorR is prerequisite to form A-LPS, and PorT is one of the T9SS component proteins. Our exhaustive random transposon mutagenesis analysis on pigmentation has revealed that 19 genes are involved in the A-LPS synthesis, 15 genes are T9SS component proteins, and 3 genes, *porX*, *porY* and *sigP* are involved in gene regulation of the T9SS component proteins^28–31^. The *porX* and *porY* genes encode a response regulator and a sensor kinase of two-component system, and the *sigP* gene encodes an ECF sigma factor^5,29^. However, more genes may relate to the pigmentation because we analyzed only non-pigmented mutants and not the slightly-pigmented mutants, which may have defects in pigmentation-related genes^31^.

A T9SS CTD-containing protein, PorA (PGN_0123), was reported to form diffuse protein bands with molecular masses of more than 35 kDa^7^ and its CTD cleavage site was determined^11^. Here we show that PorA is located in the upstream of the PorXY-SigP signaling cascade, which impacts the T9SS expression. PorA is translocated to the cell surface without the T9SS translocation machinery and localized on the cell surface in both A-LPS bound diffuse form and 23-kDa CTD-containing form, and thus PorA is a novel type of T9SS CTD-containing protein. PorA is 246 amino acids in length and is composed of an N-terminal domain and CTD with an N-terminal signal sequence consisting of 27 residues. The structure of the N-terminal domain resembles the ligand binding domain of FimH. On the basis of the structure and the results of the functional analyses, we propose a plausible mechanism for how PorA influences the T9SS expression.

## Results

### Pigmentation, hemagglutination, and gingipain activities of the *ΔporA* mutant

**Figure 1:**
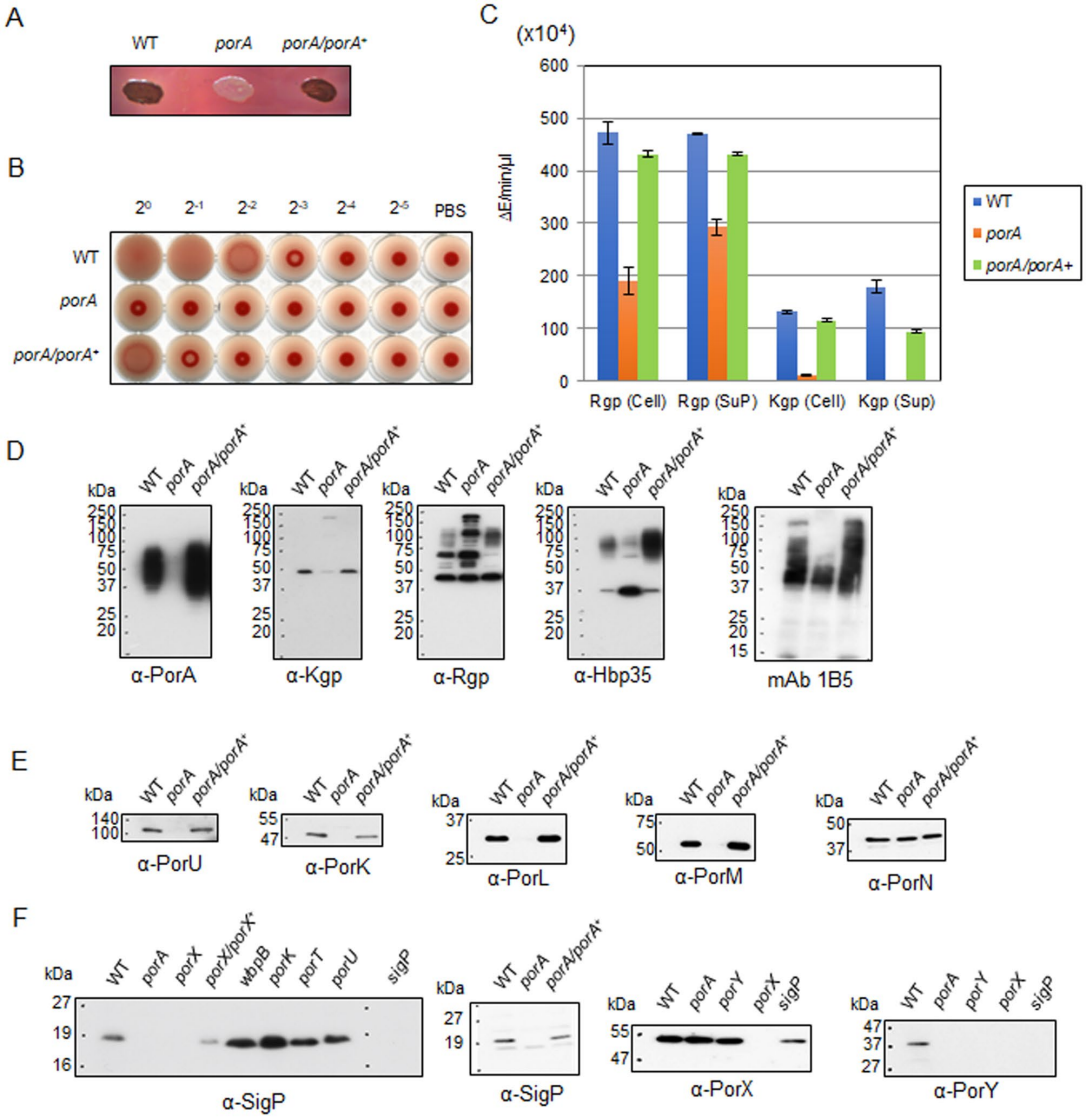
Characterization of *P. gingivalis ΔporA* mutant. (**A**) Pigmentation of the *ΔporA* mutant on blood agar plates for 3 days. (**B**) Hemagglutinating activity of the *ΔporA* mutant. (**C**) Kgp and Rgp activities of the *ΔporA* mutant. The cell lysates (cell) and vesicle-containing culture supernatants (sup) were subjected to the assay. Lane 1: wild type, Lane 2: *ΔporA*, Lane 3: *ΔporA*/*porA*^+^. (**D**) Immunoblot analyses of T9SS CTD-containing proteins and A-LPS in the *ΔporA* mutant. Cell lysates of the wild type (lane 1), *ΔporA* (lane 2), and *ΔporA*/*porA*^+^ (lane3) were analyzed by SDS-PAGE, followed by immunoblot analyses of T9SS CTD-containing proteins and A-LPS using antibodies against PorA (α-PorA), Kgp (α-Kgp), Rgp (α-Rgp) and Hbp35 (α-Hbp35), and monoclonal antibody against A-LPS (mAb 1B5). (**E**) Cell lysates of the wild type (lane 1), *ΔporA* (lane 2), and *ΔporA*/*porA*^+^ (lane3) were analyzed by SDS-PAGE, followed by immunoblot analyses of T9SS component proteins using antibodies against PorU (α-PorU), PorK (α-PorK), PorL (α-PorL), PorM (α-PorM), and PorN (α-PorN). (**F**) Immunoblot analyses of the T9SS regulatory proteins SigP, PorY, and PorX in various T9SS-related mutants. Cells of *P. gingivalis* strains were lysed with 1% *N*-Dodecyl-β-d-maltopyranoside (DDM) (vol/vol) and then subjected to SDS-PAGE, followed by immunoblot analysis using antibodies against SigP (α-SigP), PorX (α-PorX), and PorY (α-PorY). Full-length blots/gels of (**E**) and (**F**) are presented in Supplementary Figure S11.

PGN_0123, PGN_0654, and PGN_1770 are small CTD-containing proteins, but their functions are not known. We constructed deletion mutants of each protein and examined their phenotypes. The PGN_0123 mutant showed less pigmentation on blood agar plates (Fig. 1A) as expected by Klein et al.^12^, whereas PGN_0654 and PGN_1770 mutants were pigmented (Fig. S1A). Thus, we designated the gene of PGN_0123 as *porA*. The *ΔporA* mutation decreased the hemagglutinating activity and the gingipain activities of the cells (Fig. 1B,C). These functional defects were recovered by expression of *porA*^+^ from plasmid in the *ΔporA* mutant cells, indicating that PorA is required for pigmentation and virulence. In this complementation experiment, we used a shuttle vector, pTCB, and expressed the *porA* gene with the promoter of the catalase gene (*cat*) in *Porphyromonas gulae* (see in Supplemental text).

### PorA-mediated expression of T9SS component proteins

Previous studies revealed that the cause of no or less colony pigmentation is divided into two categories: one cause is defined by defects in T9SS and the other is defined by defects in A-LPS synthesis^4,27^. In the T9SS-deficient mutants, such as the *porK* and *porT* mutants, T9SS cargo proteins are not associated with A-LPS; they are detected in the periplasm only as unprocessed and unmodified forms lacking N-terminal signal peptides, and mAb 1B5, a monoclonal antibody against A-LPS, reacts to produce diffuse bands with apparent molecular masses lower than those of the wild type in SDS-PAGE^32^. On the other hand, in the A-LPS-deficient mutants such as the *porR* and *wbpB* mutants, most of the T9SS cargo proteins are secreted into culture supernatants and mAb 1B5 shows no reaction to their cell lysates^33^. To elucidate whether the PorA deficit affects the T9SS or the A-LPS synthesis, we conducted immunoblot analysis of the *ΔporA* mutant. In the *ΔporA* mutant, unprocessed and unmodified form bands of Kgp and Rgp were detected at significant amounts in addition to their mature/processed form bands, and the diffuse bands were markedly reduced for Hbp35 compared to those of the wild type and the complemented strains (Fig. 1D). The immunoblot of SDS-PAGE gels of the *ΔporA* mutant with mAb 1B5 showed diffuse bands with their apparent molecular masses lower than those of the wild type and the complemented strain (Fig. 1D). These properties of the *ΔporA* mutant were more similar to those of the T9SS-deficient mutants than the A-LPS-deficient mutants, suggesting that the *ΔporA* mutation does not affect the A-LPS synthesis but induces a partial defect in the T9SS.

We next investigated the levels of several T9SS component proteins in the *ΔporA* mutant by immunoblot analysis. PorU, PorK, PorL, and PorM were almost absent in the *ΔporA* mutant (Fig. 1E); however, PorK, PorL, and PorM were still produced at a certain level compared to corresponding null mutants (Fig. S2). In contrast, *ΔporA* mutation did not affect the amount of PorN (Fig. 1E). Since production of these Por proteins is regulated by the PorXY-SigP signaling pathway, we analyzed the amounts of PorX, PorY, and SigP in the *ΔporA* mutant (Fig. 1F). No SigP was detected in the *ΔporA* mutant, which was also the case for the *ΔporX* mutant^29^. Amounts of PorX in the *ΔporA* and *ΔporY* mutants were comparable to that of the wild type, whereas that in the *ΔsigP* mutant exhibited nearly 70% and 72% reduction in mRNA and protein levels, respectively (Fig. 1F, Fig. S3). Intriguingly, we found that no PorY protein was observed in the *ΔporA*, *ΔporX*, or *ΔsigP* mutant (Fig. 1F, Fig. S4A). qRT-PCR analysis revealed that the *porY* mRNA levels of the *ΔporA*, *ΔporX*, and *ΔsigP* mutants were reduced by 50%, 30%, and 80%, respectively, compared to that of the wild type (Fig. S4B). Therefore, marked decrease of the PorY protein in the *ΔporA*, *ΔporX*, and *ΔsigP* mutants may be caused not only at the mRNA level but also at the protein level.

### Analysis of gain-of-function mutants from the *ΔporA* mutant

**Figure 2:**
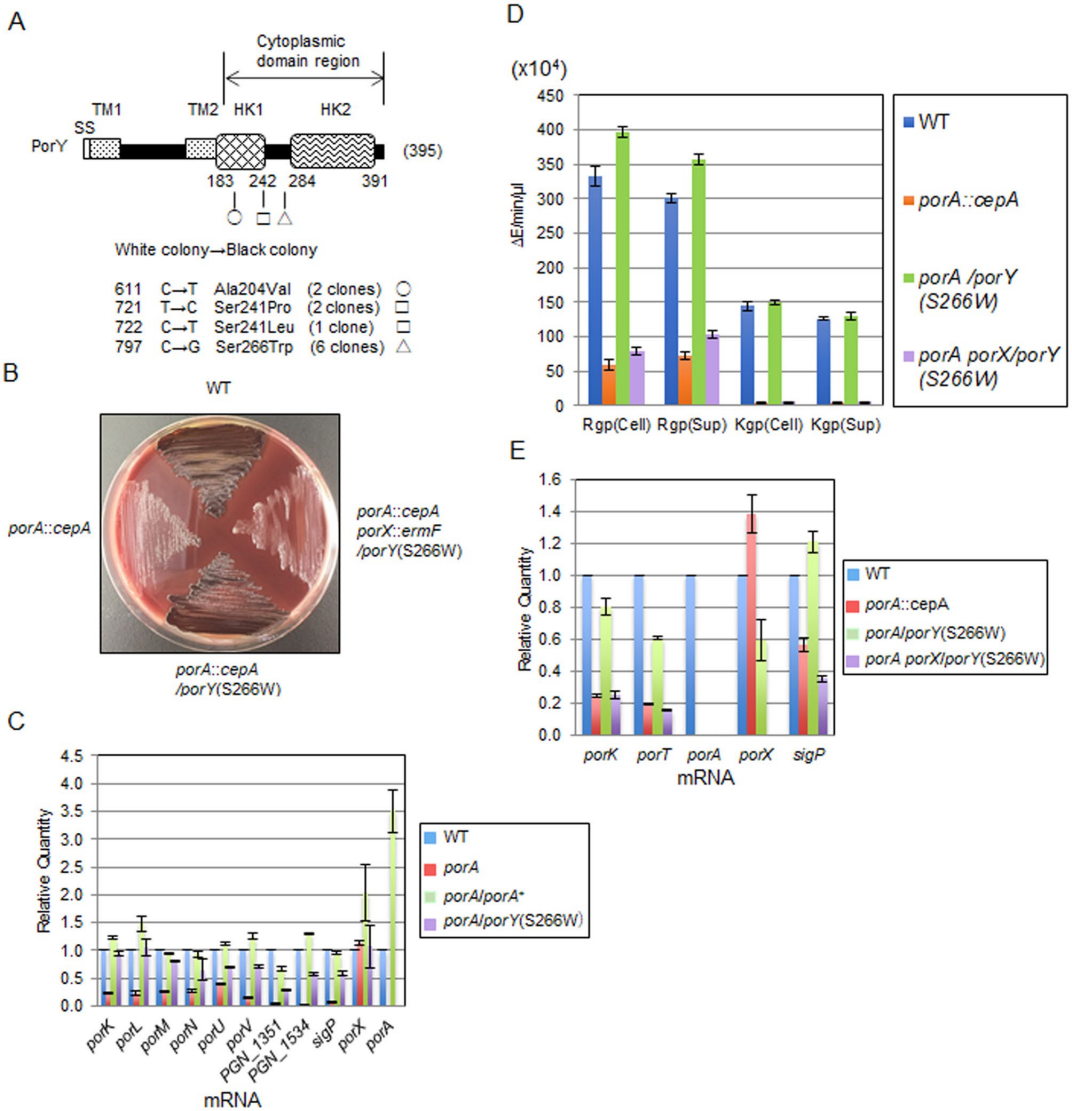
Pseudo-revertants from the *porA* deletion mutant. (**A**) Mutations in *porY* of the pseudo-revertants. Mutations causing amino acid substitutions were found in the cytoplasmic domain-encoding region of *porY*. HK, histidine kinase domain; SS, signal sequence; TM, transmembrane region. Circle, square, and triangle indicate amino acid substitutions at 204, 241, and 266, respectively. (**B**) Colony pigmentation of the wild type, *ΔporA*, *ΔporA*/*porY*(S266W), and *ΔporA ΔporX*/*porY*(S266W) strains on the blood agar plate for 6 days. (**C**) qRT-PCR expression analysis of various T9SS-related genes in the wild type, *ΔporA*, *ΔporA/porA*^+^ and *ΔporA*/*porY*(S266W) strains. The mean of expression of each wild type gene was regarded as 1. (**D**) Gingipain activities of the wild type, *ΔporA*, *ΔporA*/*porY*(S266W), and *ΔporA ΔporX*/*porY*(S266W) strains. (**E**) Expression of the *porK*, *porT*, *porA*, *porX*, and *sigP* genes in the wild type, *ΔporA*, *ΔporA*/*porY*(S266W), and *ΔporA ΔporX*/*porY*(S266W) strains. RNA samples of the strains were subjected to qRT-PCR analysis. The mean expression of each wild type gene was regarded as 1.

We isolated gain-of-function mutants from the *ΔporA* mutant by repeating inoculation of the *ΔporA* mutant on blood agar plates. Black-pigmented colonies appeared after three times successive inoculation. Two pseudo-revertants were subjected to genome analysis. Both of the two strains had the same two mutations that caused the conversion of the 241st Ser of PorY and the 295th Val of PGN_1053 (putative phospho-2-dehydro-3 deoxyheptonate aldolase/chorismate mutase) to Leu and Glu, respectively. These pseudo-revertants appeared to be siblings, so that we accounted the two strains as one clone (Fig. 2A). Next, we sequenced the *porY* gene regions from ten pseudo-revertants that were obtained independently, and found that all the strains possessed amino acid-substituted mutations in the putative cytoplasmic domain region of the *porY* gene (Fig. 2A). To confirm whether the *porY* mutations compensate the defects of the *ΔporA* mutant, the *porY* gene carrying one of the mutations, *porY*(S266W), was introduced into the *ΔporA* mutant by a shuttle plasmid. The resulting strain *ΔporA*/*porY*(S266W) showed a black-pigmented phenotype, as did the wild type (Fig. 2B). PorY is a component of the two-component signal transduction system involved in gene regulation of the T9SS component proteins^28–31^. PorY is a sensor kinase and PorX is a response regulator of the two-component system. The PorXY system positively regulates the T9SS expression by influencing SigP*,* an extra-cytoplasmic function (ECF) sigma factor^5,29^. Taken together with marked decrease of PorY and SigP in the *ΔporA* mutant, we speculated that PorA influences the expression of the T9SS related genes at mRNA level through the PorXY-SigP signaling pathway. To confirm this hypothesis, qRT-PCR analysis of the *ΔporA* mutant was performed. The *ΔporA* mutant showed markedly reduced mRNA levels of *PGN_1351*, *PGN_1534*, and T9SS component genes such as *porK*, *porL*, *porM*, *porN*, *porU*, and *porV* (Fig. 2C), all of which are reported to be down-regulated in the *porX* and *sigP* mutants^29^. Notably, the mRNA level of *sigP* was also markedly decreased in the *ΔporA* mutant compared to that of the wild type. Complemented strain *ΔporA*/*porA*^+^ and strain *ΔporA/porY*(S266W) recovered the expression of all these genes to the wild type level. We also confirmed that the *ΔporA/porA*^+^ strain recovered the SigP protein expression to the wild type level (Fig. 1F). To examine whether the *ΔporA/porY*(S266W) strain still utilizes the PorXY-SigP signaling pathway for T9SS regulation, a *porX* mutation was introduced into the chromosome of the *ΔporA/porY*(S266W) strain. The resulting strain *ΔporA ΔporX*/*porY*(S266W) showed a non-pigmented phenotype, indicating that PorY(S266W) also requires PorX for the phenotypic recovery (Fig. 2B,D,E). Furthermore, we constructed a *ΔporA ΔporY* mutant and introduced the *porY*(S266W) gene-carrying shuttle plasmid to the *ΔporA ΔporY* mutant and found that the resulting *ΔporA ΔporY*/*porY*(S266W) strain shows pigmented, produces PorY(S266W), and has Rgp and Kgp activities (Figs. S1B, S5, and S6). In addition, the *ΔporA ΔporY* mutant was stably non-pigmented after several passages and exhibited significantly reduced Kgp activity (Figs. S1B, S5, and S6). These results suggest that PorA is located upstream of the PorXY-SigP signaling pathway.

### Translocation of PorA onto the cell surface in a T9SS translocation machinery-independent manner

**Figure 3:**
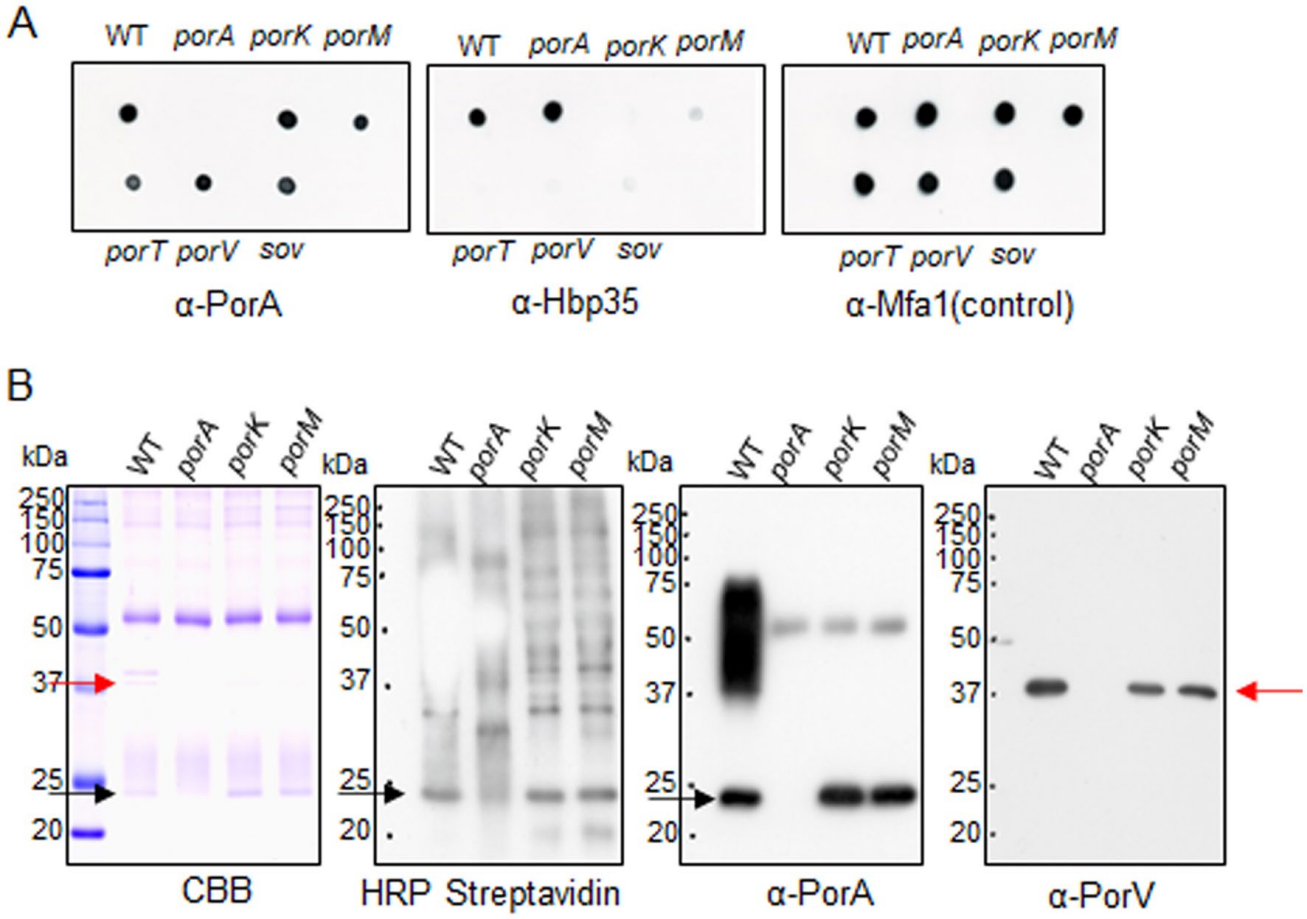
Cell surface localization of PorA in *P. gingivalis* cells. (**A**) Cells of *P. gingivalis* wild type, *ΔporA, porK*, *porM*, *porT*, *porV*, and *sov* strains were blotted on nylon membranes and immunodetected by antibodies against PorA (α-PorA), Hbp35 (α-Hbp35), and Mfa1 (α-Mfa1). (**B**) Biotin-labeled cells of *P. gingivalis* wild type, *ΔporA, porK*, and *porM* strains were lysed and then immunoprecipitated by α-PorA. The immunoprecipitated samples were subjected to SDS-PAGE, followed by immunoblot analyses using HRP conjugated streptavidin, α-PorA, and antibody against PorV (α-PorV). CBB: Coomassie Brilliant Blue staining. Red and black arrows indicate PorV and PorA proteins, respectively.

Since the C-terminal amino acid sequence of PorA (D163-K246) shows significant homology to the T9SS CTD sequence^6^, PorA is expected to be secreted to the cell surface through the T9SS. We therefore examined the PorA exposure on the cell surface by a dot blot analysis of intact cells with anti-PorA antibody (α-PorA) and with anti-Hbp35 antibody (α-Hbp35) reacting to Hbp35, a typical group I T9SS cargo protein. The wild-type cells reacted to both α-PorA and α-Hbp35, suggesting that PorA is present on the cell surface (Fig. 3A). α-Hbp35 did not react to the cell surface of T9SS-deficient mutant (*porK*, *porM*, *porT*, *porV*, or *sov*), whereas α-PorA reacted to those of the T9SS-deficient mutants (Fig. 3A)^15^. The result suggests that PorA can be translocated on the cell surface through a pathway without the T9SS translocation machinery. Hbp35 was detected on the cell surface in the *ΔporX* and the *ΔporY* mutants as well as the *ΔporA* mutant, but not in the *porK* mutant, indicating that the phenotype of the *ΔporA* mutant is similar to those of the *ΔporX* and *ΔporY* mutants (Fig. S7).

Cell surface exposure of PorA was further examined by biotin labelling assay. Proteins exposed on the cell surfaces of *porK, porM, ΔporA* mutant and the wild-type cells were labeled with biotin. The cells were lysed, and the lysates were immunoprecipitated with α-PorA. The immunoprecipitated samples were loaded in SDS-PAGE and immunodetected with HRP conjugated streptavidin (HRP-S) and α-PorA (Fig. 3B). The sample from the wild-type cells showed diffuse bands and a thick band of about 23 kDa in both HRP-S and α-PorA staining gels. The diffuse bands are typical for the group I cargo proteins covalently bound to A-LPS on the cell surface. The theoretical molecular mass of PorA without the N-terminal signal sequence is 23,426. Therefore, these results suggest that PorA is located on the cell surface in two forms, an A-LPS bound form and a 23-kDa CTD-containing form. On the other hand, the *porK* and *porM* mutant samples showed the 23-kDa band but no diffuse bands in the HRP-S and α-PorA staining gels, indicating that PorA is present only in the 23-kDa CTD-containing form on the cell surfaces of the T9SS-deficient mutants.

The PorV protein can bind several T9SS CTD-containing proteins^34,35^. Glew et al.^35^ reported interaction between PorA and PorV. To confirm the interaction, the samples immuno-precipitated with α-PorA were subjected to immunoblot analysis with α-PorV. A protein band with 37 kDa reacted to α-PorV, indicating interaction of PorA and PorV (Fig. 3B).

### Interaction of CTD of PorA with PorV

**Figure 4:**
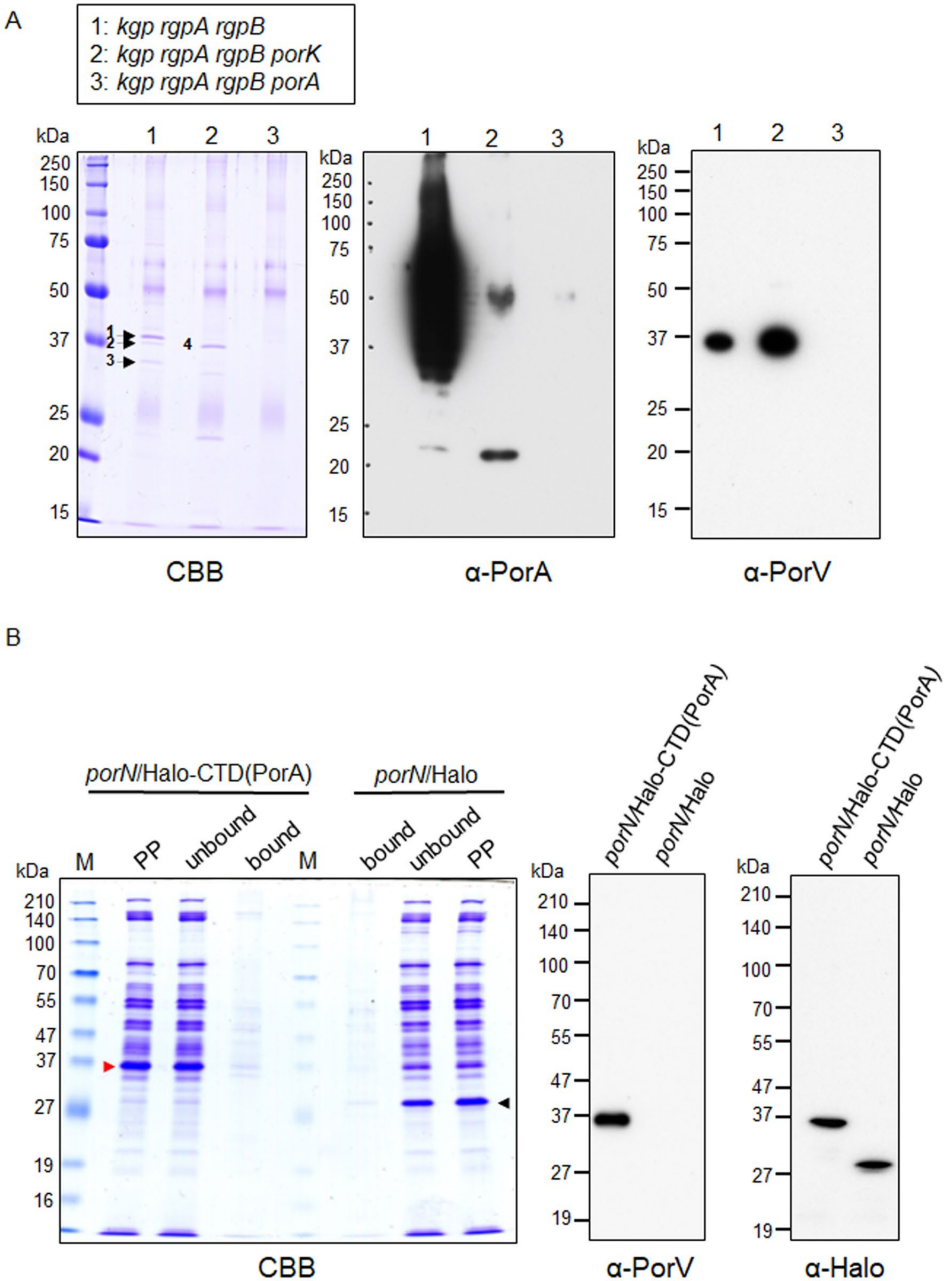
Interaction between PorA and PorV. (**A**) Immunoblot analysis with α-PorA and α-PorV. The α-PorA-immunoprecipitated proteins from the membrane fractions of *P. gingivalis* cells treated with a chemical crosslinker, dimethyl 3,3′-dithiobispropionimidate (DTBP) were separated by SDS-PAGE, followed by immunoblot analyses using α-PorA and α-PorV. Protein bands in the CBB-stained gel indicated by numerals were subjected to peptide mass fingerprinting analysis (Table 1). CBB: Coomassie Brilliant Blue staining. (**B**) Immunoblot analysis of Halo-CTD(PorA) chimera protein fraction with α-PorV. Cells of the *porN* mutants expressing Halo-CTD(PorA) chimera protein (*porN*/Halo-CTD(PorA)) and Halo protein (*porN*/Halo), respectively, were treated with DTBP. Partially purified samples (PP) were obtained from the cells and added to HaloLink Resin. After centrifugation, the supernatants (unbound) were removed and the resin was washed three times with HaloTag purification buffer. After centrifugation, the resin was dissolved with SDS sample buffer containing dithiothreitol (bound). The resulting ‘bound’ samples from *porN*/Halo-CTD(PorA) and *porN*/Halo were heat denatured and subjected to SDS-PAGE and immunoblot analyses with α-PorV and antibody against Halo tag (α-Halo). CBB: Coomassie Brilliant Blue staining. Red and black arrowheads indicate Halo-CTD(PorA) and Halo proteins, respectively.

We performed peptide mass fingerprinting to identify proteins immuno-precipitaed with α-PorA. In the experiment, we used derivatives of a gingipain-null mutant (*kgp rgpA rgpB*) to avoid excessive proteolysis during sample preparation. Membrane fractions of *kgp rgpA rgpB*, *kgp rgpA rgpB porK*, and *kgp rgpA rgpB ΔporA* cells treated with a chemical crosslinking reagent, dimethyl 3,3′-dithiobispropionimidate (DTBP) were prepared, immuno-precipitated with α-PorA and subjected to SDS-PAGE. Protein bands on the gel indicated by numerals were subjected to peptide mass fingerprinting using MALDI TOF–MS (Fig. 4A and Table 1). The gel was also subjected to immunoblot analyses with α-PorA and α-PorV (Fig. 4A). The sample of *kgp rgpA rgpB* showed diffuse bands and a weak band with 23 kDa that reacted to α-PorA, and a band with 37 kDa that reacted to α-PorV, while the sample of *kgp rgpA rgpB porK* showed relatively strong bands with 23 kDa and 50 kDa that reacted to α-PorA and a band with 37 kDa that reacted to α-PorV. The 50-kDa band reacting to α-PorA appeared to be non-specific because the *ΔporA* and *kgp rgpA rgpB ΔporA* mutants also showed the 50-kDa band (Figs. 3B and 4A). However, we did not find any PorA-PorV heterodimer covalently cross-linked by treatment with DTBP even at non-reduced conditions, suggesting that the DTBP treatment did not work between PorA and PorV. Therefore, we performed the immuno-precipitation analysis with α-PorA in the absence of DTBP to examine whether there was stable interaction between PorA and PorV. Membrane fractions of the wild-type, *porK*, and *ΔporA* cells were prepared, immuno-precipitated with α-PorA, and subjected to SDS-PAGE and native PAGE, and the following immunoblot analyses with α-PorA and α-PorV (Fig. S8). The SDS-PAGE analysis without the DTBP treatment showed essentially the same results as that with the DTBP treatment (Fig. 4A, Fig. S8A). The native PAGE analysis showed diffuse protein bands with high molecular masses that reacted to both α-PorA and α-PorV in the samples of wild-type and *porK* cells (Fig. S8B). These results suggest that the 23-kDa CTD-containing PorA interacts with PorV.

**Table 1:** Peptide mass fingerprinting (PMF) analysis.

Strain	Band	Gene	Annotation	Mascot score
*kgp rgpA rgpB*	1	PGN_0728	OmpA	43
*kgp rgpA rgpB*	2	PGN_0023	PorV	87
*kgp rgpA rgpB*	3	PGN_1484	Methylated-DNA-protein-cysteine methyltransferase	59
*kgp rgpA rgpB porK*	4	PGN_0023	PorV	61

Proteins in the bands with numerals in the CBB stained gel of Fig. 4A were identified by PMF analysis using MALDI TOF–MS.

To determine whether the PorV protein can bind the CTD of PorA, we constructed a gene encoding a chimera protein consisting of the N-terminal signal peptide of Kgp, the Halo tag, and the CTD of PorA and expressed it in the *porN* mutant. The chimera protein was purified using HaloLink Resin after protein crosslinking with DTBP and subjected to SDS-PAGE and immunodetection (Fig. 4B). The result suggested that PorV can bind the PorA CTD. Furthermore, we examined whether the CTDs of other T9SS cargo proteins interact with PorV using the same method. The results showed that the CTDs of RgpB, Mfa5, PPAD, and Kgp interacted with PorV although the strength of interaction was different one another, and the CTD of Hbp35 did not (Fig. S9).

### Formation of the PorA A-LPS-bound form in co-culture of the *porK* and *porR* mutants

**Figure 5:**
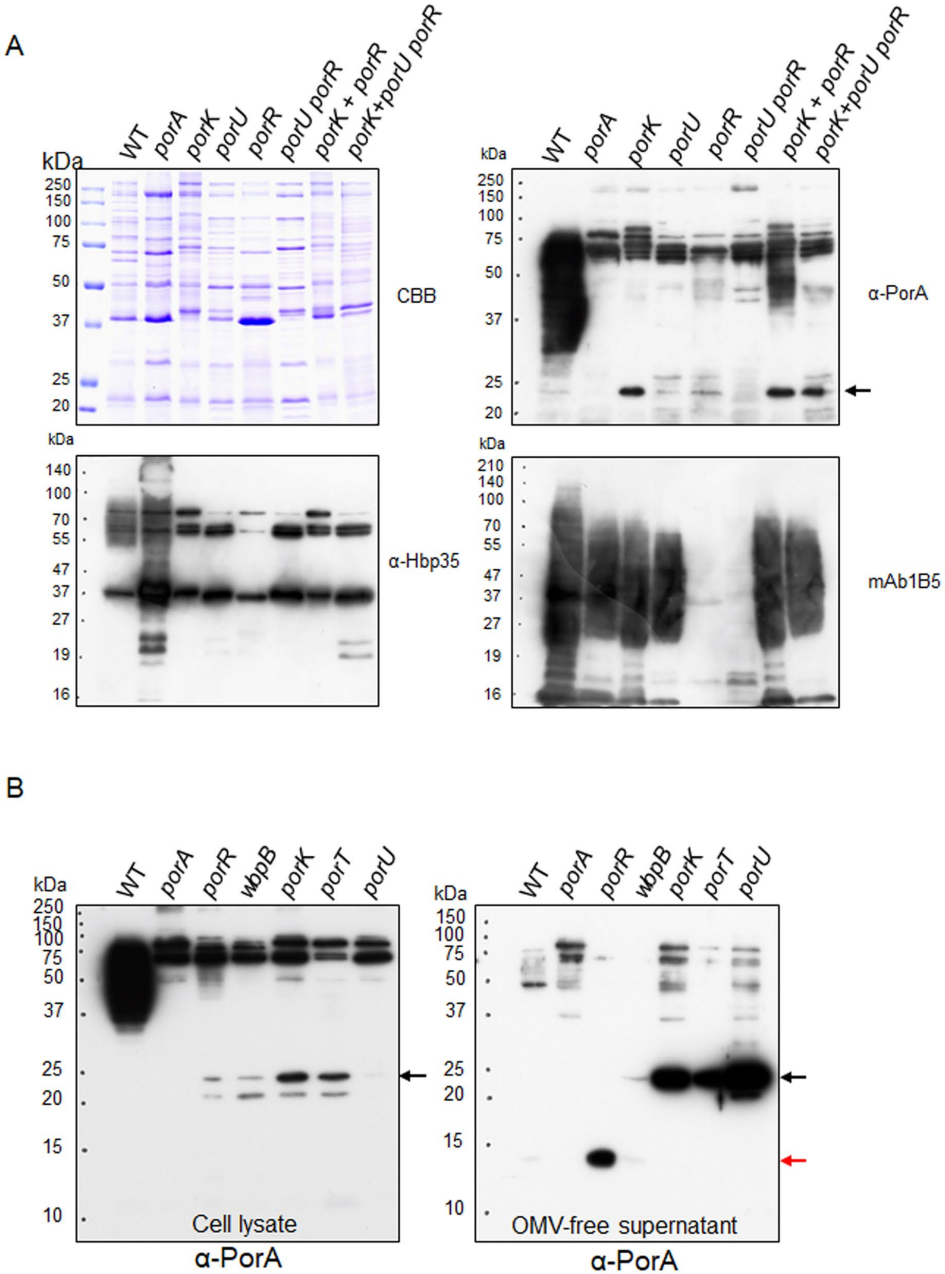
Appearance of A-LPS-bound PorA in co-culture of the *porK* and *porR* mutants. (**A**) OMV fractions of cultures of the wild type, *ΔporA*, *porK*, *porU*, *ΔporR*, and *porU ΔporR* strains and co-cultures of the *porK* and *ΔporR* strains, and the *porK* and *porU ΔporR* strains were subjected to SDS-PAGE, followed by immunoblot analyses using α-PorA, α-Hbp35, and mAb 1B5. CBB: Coomassie Brilliant Blue staining. The black arrow indicates the 23-kDa CTD-containing PorA. (**B**) Cell lysates (left) and OMV-free culture supernatants (right) of the wild type, *ΔporA*, *ΔporR*, *wbpB*, *porK*, *porT*, and *porU* strains were subjected to SDS-PAGE, followed by immunoblot analysis with α-PorA, respectively. The black and red arrows indicate the 23-kDa CTD-containing PorA and the 14-kDa CTD-lacking PorA, respectively.

Group I CTD cargo proteins are covalently bound to A-LPS following the cleavage of their CTDs by the PorU sortase secreted to the cell surface through the T9SS. We analyzed whether the 23-kDa CTD-containing PorA that is translocated to the cell surface without passing through the T9SS translocation machinery in the *porK* mutant can be bound to A-LPS after removal of CTD by PorU. The *porK* mutant cells were mixed with cells of a *ΔporR* mutant and the mixture was co-cultured for two days. PorK is an essential component of the T9SS translocation machinery, so the *porK* mutant has no T9SS, but produces A-LPS. PorR is an enzyme involved in A-LPS synthesis, so the *ΔporR* mutant does not synthesize A-LPS, but has normal T9SS including PorU on the cell surface. If the 23-kDa CTD-containing PorA on the cell surface can be a substrate of PorU, the CTD of PorA in the *porK* mutant would be cleaved and the remaining part of PorA would be bound to A-LPS by PorU in the *ΔporR* mutant. Such reaction may take place between the outer membrane vesicles (OMV) of the *porK* and *ΔporR* mutants. OMV fractions of the culture were subjected to SDS-PAGE followed by immunoblot analysis with α-PorA (Fig. 5A). Diffuse bands appeared in the OMV fraction of the co-culture of the *porK* and the *ΔporR* mutants, but no such pattern was detected in that of the individual culture of the mutants and in that of the co-culture of the *porK* and the *porU ΔporR* mutants. In contrast, the OMV fraction of the co-culture of the *porK* and the *ΔporR* mutants showed no diffuse bands with α-Hbp35 because Hbp35 is not secreted on the cell surface without the T9SS. These results also suggest that the 23-kDa CTD-containing PorA is present on the cell surfaces of the T9SS-deficient mutants and can be converted to the A-LPS-bound diffuse form in the presence of PorU. In this analysis, we found that there was a markedly reduced amount of 23-kDa CTD-containing PorA in the OMV fraction of the *porU* mutant compared to that of the *porK* mutant (Fig. 5A). The amount of the 23-kDa CTD-containing PorA was almost equivalent to that in the OMV fraction of the wild type. The wild type had a large amount of A-LPS-bound diffuse PorA. Next, we examined the presence and location of PorA in the *porU* mutant (Fig. 5B). The cell lysate samples showed the results similar to those of the OMV samples, whereas the *porU* mutant showed a large amount of the 23-kDa CTD-containing PorA in the OMV-free culture supernatant samples.

The *porK* mutant, which created the 23-kDa CTD-containing PorA but not the A-LPS-bound diffuse PorA on the cell surface (Fig. 3B), produced SigP equal or higher levels than the wild type (Fig. 1F). SigP produced in the *porK* mutant appears to be functional because the mutant showed expression of SigP-regulated genes at both mRNA and protein levels (Fig. S10). These results suggest that at least the 23-kDa CTD-containing PorA on the cell surface has the ability to activate the PorXY-SigP signaling pathway of the T9SS expression to yield the functional SigP.

### Crystal structure of the N-terminal domain of PorA

**Figure 6:**
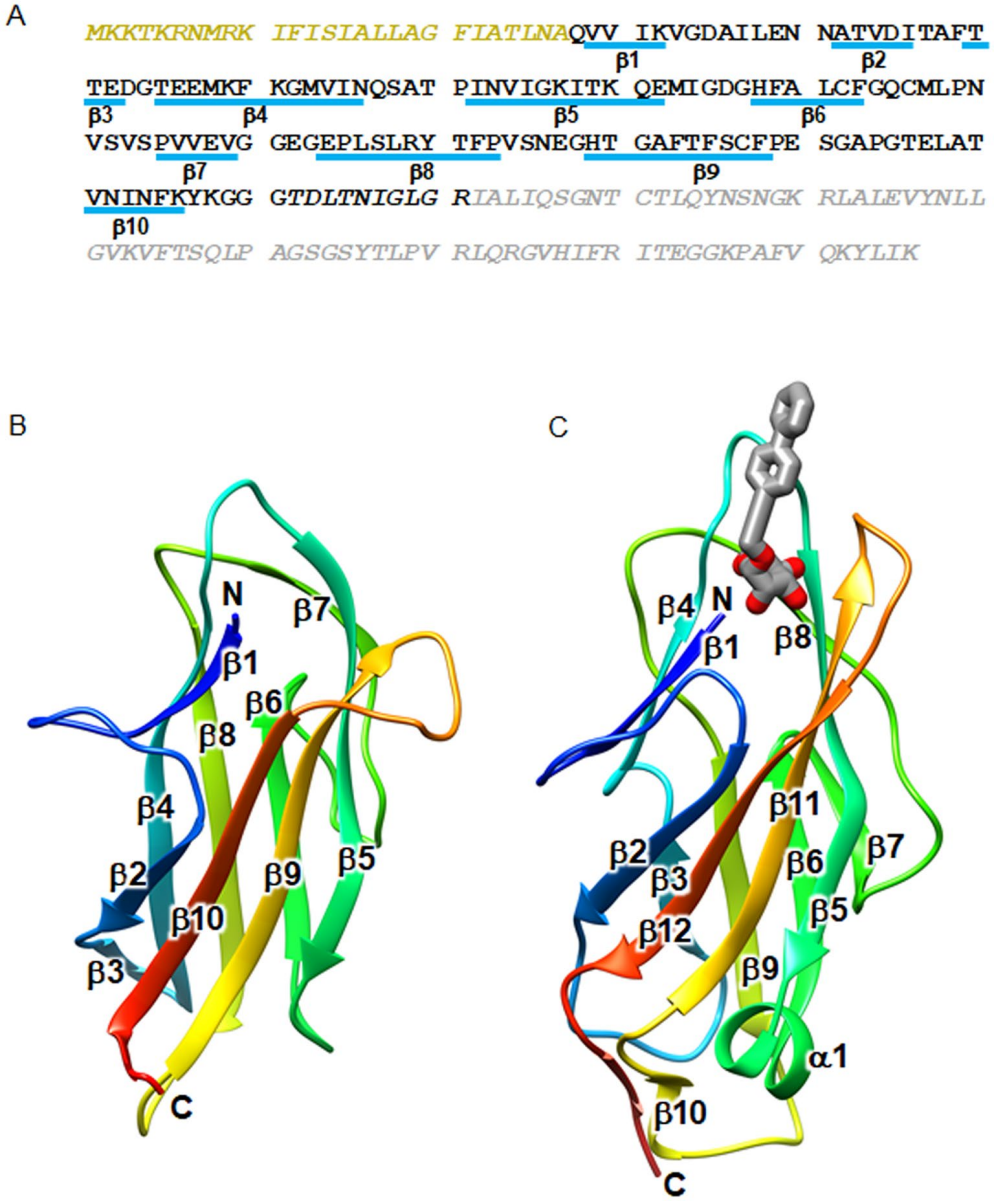
Crystal structure analysis of PorA. (**A**) Amino acid sequence of PorA. The core fragment used for crystallization is shown in black and the region removed by protease is in gray. The signal peptide region is colored in yellow. β-strands are indicated by blue bars with labels. The residues not included in the structure model are shown in italics. (**B**) Structure of the core fragment of PorA. The model is color coded from blue to red from the N- to the C-terminus. (**C**) Structure of the mannose-binding lectin domain of FimH in complex with propynyl biphenyl α-d-mannoside (PDB ID: 4av5). The model is color coded from blue to red from the N- to the C-terminus. The ligand molecule is drawn in stick model with oxygen atoms colored red and carbon atoms colored gray.

We next conducted the X-ray crystal structure analysis of PorA. Initially, we tried to determine the structure of the 23-kDa CTD-containing PorA, but no crystal was obtained. Therefore, we determined the core region of PorA by limited proteolysis of purified PorA with trypsin. The 23-kDa CTD-containing PorA was digested into a fragment composed of Q28 to R171 (Fig. 6A), and the C-terminal 75 residues, which corresponds to CTD, were degraded into small peptides. Thus, we purified and crystallized the trypsin-resistant fragment, termed PorA-N. The structure of PorA-N was determined at 1.3 Å resolution (Fig. 6B). The C-terminal 10 residues were invisible in the electron density map, and therefore, residues Q28-G161 were modeled. The crystal belongs to the space group *C*2 and contains a single PorA-N molecule in an asymmetric unit. The structure of PorA-N adopts an immunoglobulin-like fold composed of 10 β-strands (Fig. 6B). The Dali database search revealed that FimH from *Escherichia coli* in complex with propynyl biphenyl α-d-mannoside, a ligand analogue, gave the best structural similarity (PDB ID: 4av5, Z-score: 8.4) to PorA-N (Fig. 6C). FimH is a tip protein of Type 1 pilus and binds to mannose. This structural similarity suggests that PorA-N may bind an unknown molecule. The mannose binding site of FimH is located at the top of the barrel, and the N-terminus, the loop connecting β4 and β5 and the loop connecting β11 and β12 (the 11–12 loop) surround the ligand and form hydrogen bonds with the ligand. In contrast, the barrel top of PorA-N shows a wide-open structure. The loop connecting β9 and β10 corresponding to the 11–12 loop of FimH extends outward. Thus, the barrel top of PorA-N is exposed to the outside, implying that interaction with the unknown binding partner may induce a structural change of PorA-N.

## Discussion

We found in this study that PorA impacts the PorXY-SigP signaling pathway that regulates expression of the T9SS in *P. gingivalis*. PorA is the first T9SS CTD-containing protein involved in gene regulation of the T9SS component proteins. Like other T9SS CTD-containing proteins, PorA is secreted on the cell surface. Previously characterized T9SS CTD-containing proteins such as Hbp35 are not secreted on the cell surface in the absence of the T9SS secretion machinery. In contrast, PorA is located on the cell surface of the T9SS-deficient mutants (*porK*, *porM*, *porT*, *porV* and *sov*) as its CTD-containing form (Fig. 3A). Lauber et al.^36^ reported that SprA, a Sov homolog of *Flavobacterium johnsoniae*, forms a water-filled conduit of sufficient size to allow the passage of folded proteins across the outer membrane. Although most T9SS CTD-containing proteins are secreted via the T9SS in *F. johnsoniae*, a small CTD-containing protein with 112 amino acid residues, Fjoh_0547, is secreted in the culture supernatants of *sprA* and *porV* mutants^36^, suggesting that Fjoh_0547 can be secreted without passing through the SprA channel. In this context, PorA, the second smallest CTD-containing protein (246 amino acid residues) in *P. gingivalis*, may be translocated to the cell surface through a similar pathway as Fjoh_0547 without passing through the Sov channel.

**Figure 7:**
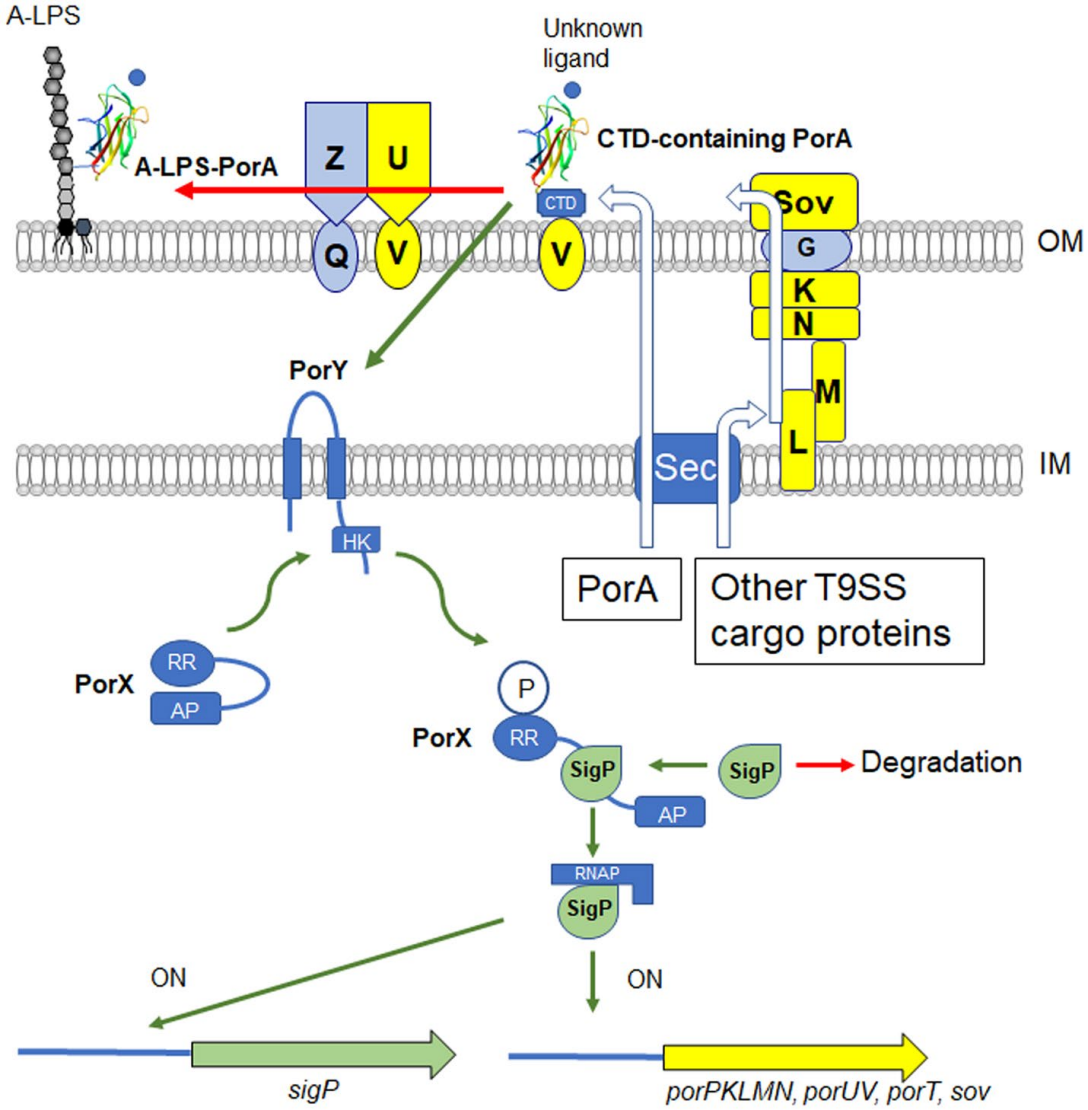
Proposed model of regulation of the T9SS expression in *P. gingivalis*. The PorA protein is located in the outer membrane. In the proposed model, the 23-kDa CTD-containing PorA activated by an unknown ligand activates the PorY sensor kinase of the PorXY two-component system directly or indirectly. Alternatively, the 23-kDa CTD-containing PorA may activate PorY without any environmental substances. The PorA-activated PorY activates the PorX response regulator. The PorY-activated PorX activates the SigP sigma protein and the resulting active SigP upregulates its own gene and several T9SS component genes.

Previously characterized T9SS cargo proteins belong to one of the three groups; I, II and III (Table 2). CTDs of groups I and II proteins are removed by PorU. A-LPS is then covalently bound to the group I proteins, but not to the group II proteins. Group III proteins retain their CTDs on the cell surface and do not bind A-LPS. PorA has both characteristics of groups I and III. PorA is present on the cell surface in both the A-LPS bound diffuse form and the 23-kDa CTD-containing form. Thus, PorA is a novel type of T9SS CTD-containing protein. *P. gingivalis* T9SS consists of the translocation machinery (PorG, PorK, PorL, PorM, PorN, and Sov), the attachment complex (PorQ, PorU, PorV, and PorZ), the PorV shuttle protein, and other components with unknown functions (PorE, PorF, PorP, PorT, and PorW) (Fig. 7)^37^. PorA appears to use the PorV shuttle protein and the attachment complex for covalently binding to A-LPS, but not to use the T9SS translocation machinery for secretion to the cell surface.

**Table 2:** T9SS cargo proteins in *P. gingivalis*.

PGN_number	PG_number	Annotation	Amino acids^a^	Group^b^	References
0014^c^	0018^c^	Hypothetical protein	748/748	ND	
0022	0026	PorU	1158/1158	III	^11^
0123	2172	PorA	246/248	I and III	This study, ^12^
0152	2102	TapA, immunoreactive 61 kDa antigen PG91	540/540	I	^13^
0291	0182	Mfa5, von Willebrand factor type A domain protein	1288/1226	II	^14^
0295^c^	Lack	C-terminal domain of Arg- and Lys-gingipain proteinase	293/–	ND	
0335	0232	Cpg70, zinc carboxypeptidase	821/821	I	^15^
0458^c^	Lack	Hypothetical protein	508/–	ND	
0509	1604	PorZ, immunoreactive 84 kDa PG93	776/776	III	^16^
0561	1548	PrtT, trypsin like proteinase	840/frameshift	ND	
0654	0611	Putative lipoprotein	313/316	ND	This study
0657	0614	Hypothetical protein	308/326	ND	
0659	0616	Hbp35	344/344	I	^15,17^
0693	0654	Hypothetical protein	390/390	ND	
0795	0769	Fibronectin type III domain protein	713/540	ND	
0852	1374	Immunoreactive 47 kDa antigen PG97 LRR protein	428/428	ND	
0898	1424	PPAD, peptidylarginine deiminase	556/556	I	^18^
0900	1427	Periodontain, thiol protease/hemagglutinin PrtT precursor	843/843	ND	
1115	1326	Hemagglutinin, putative	369/377	ND	
1317^c^	1035^c^	Hypothetical protein	496/496	ND	
1321	1030	Hypothetical protein	446/446	ND	
1416	0553	PepK, extracellular protease	940/940	I	^19^
1466	0506	RgpB, arginine-specific cysteine proteinase	736/736	I	^6^
1476	0495	Hypothetical protein	464/469	ND	
1556	0411	Hemagglutinin, putative	925/925	ND	
1611	0350	Internalin-related protein LRR protein	485/484	ND	
1728	2024	Kgp, lysine-specific cysteine proteinase	1723/1706	II	^2^
1733	1837	HagA, hemagglutinin protein	2628/2164	II	^2^
1767	1798	Immunoreactive 46 kDa antigen PG99 RHS repeat protein	423/405	ND	
1770	1795	Hypothetical protein	262/273	ND	This study, ^12^
1817^c^	Lack	Hypothetical protein	145/–	ND	
1970	2024	RgpA, hemagglutinin protein HagE	1703/1706	II	^20,21^
2065	2198	Immunoreactive 32 kD antigen PG25	293/293	ND	
2080	2216	Hypothetical protein	576/576	ND	^12^
Lack	0183	Lipoprotein, putative	–/2204	ND	
Lack	0410	Hypothetical protein gingipain-like peptidase C25	–/1294	ND	
Lack	0626	Hypothetical protein	–/288	ND	
Lack	1102^c^	Hypothetical protein	–/925	ND	
Lack	1969	Hypothetical protein	–/300	ND	
Lack	2100	TapC, immunoreactive 63 kDa antigen PG102	–/554	ND	

^a^Left and right numbers indicate amino acids from ATCC 33277 and W83, respectively.

^b^Group I indicates that C-terminal domain is cleaved and then processed protein forms diffuse bands in SDS-PAGE. Group II indicates that C-terminal domain is cleaved but processed protein does not form diffuse bands in SDS-PAGE. Group III indicates that C-terminal domain is not cleaved. ND, not determined.

^c^Not reported in Lasica et al.^8^.

The *porA* deletion mutant formed non-pigmented colonies on the blood agar plate, decreased hemagglutination and gingipain activities, and reduced expression of T9SS component genes such as *porK*, *porL*, *porM*, and *porU*, indicating that the *ΔporA* mutant is deficient in the T9SS. However, the *ΔporA* mutant is not a T9SS-null mutant, but still expresses a small amount of T9SS component proteins. We detected Hbp35 on the cell surface of the *ΔporA* mutant. This phenotype is very similar to the *ΔporX* and *ΔporY* mutants, supporting that PorA influences the PorXY-SigP signaling pathway.

PorY and PorX proteins are the sensor histidine kinase and the response regulator of the two-component system, respectively, which regulates the T9SS^29^. What activates the PorY sensor kinase remains to be determined. Since the amino acid substitution in the cytoplasmic domain of PorY compensated the defective phenotype of the *ΔporA* mutant, it is plausible that PorA activates the PorY sensor kinase. In our preliminary experiments, however, direct interaction between PorA and PorY was not detected. PorA is located on the cell surface whereas PorY is in the inner membrane, so other factor(s) should mediate between PorA and PorY.

ECF sigma factor was first recognized as a distinct subgroup of σ^70^-like factor in 1994^38^ and after that it has been found in many bacterial species^39^. In most cases, ECF sigma factor is regulated by its cognate anti-sigma factor protein, which is encoded by a downstream gene on the same transcriptional unit^39^. In *P. gingivalis*, there are six ECF sigma factors such as PGN_0274 (SigP)^29,40^, PGN_0319 (SigCH)^41^, PGN_0450, PGN_0970, PGN_1108^42^, and PGN_1740 (SigH)^43^. Their downstream CDSs such as PGN_0320, PGN_0451, PGN_0969, and PGN_1107 may encode anti-sigma factors; however, it has not been elucidated whether they are anti-sigma factors. SigP regulates gene expression of the T9SS components^29^. PorX binds SigP but not DNA^29^. PorX has the PglZ domain, which may function as a phosphatase, phosphomutase, or phosphodiesterase^44^, in its C-terminal region. Thus, PorX activated by PorY binds and activates SigP. The *sigP* gene is auto-regulated by its own gene product, SigP^40^. We previously found that the *sigP* gene was down-regulated in the *ΔporX* mutant^29^. This may be because the PorX-activated SigP, not the intact SigP, induces the expression of the *sigP* gene. In this study, down-regulation of the *sigP* gene was also found in the *ΔporA* mutant, that is consistent with the idea that PorA is located upstream of the PorXY-SigP signaling pathway (Fig. 7).

Most of T9SS cargo proteins share a common structural architecture composed of a signal peptide, a functional domain(s), an Ig-like domain and CTD^45,46^. The role of the Ig-like domain is believed to stabilize the functional domain^46^. However, PorA is composed of a signal peptide, the Ig-like domain and CTD. The Ig-like domain structure of PorA differs from those of other T9SS cargo proteins, such as Hbp35, Kgp and Rgp, but resembles the mannose-binding lectin domain of FimH. The structural similarity to FimH suggests that on the cell surface PorA binds an unknown environmental substance that stimulates PorA to impact the PorXY-SigP signaling pathway.

There are two forms of PorA on the cell surface: the A-LPS-bound diffuse form and the 23-kDa CTD-containing form. Production of functional SigP in mutants deficient in T9SS components such as the *porK* and *porT* mutants suggests that the 23-kDa CTD-containing PorA on the cell surface has the ability to activate the PorXY-SigP pathway. The 23-kDa CTD-containing PorA is cleaved by the PorU protease in the attachment complex and the resulting mature/processed PorA is covalently bound to A-LPS. If the A-LPS-bound diffuse form has no ability to activate the PorXY-SigP pathway, the attachment complex would determine amounts of PorA with the activating ability. Because PorU and PorZ in the attachment complex are secreted by the T9SS translocation machinery, the attachment complex on the cell surface is a reflection of the functionality of the T9SS translocation machinery^37^. This may imply the following feedback system in the regulation of the T9SS: When operation of the T9SS translocation machinery decreases, amounts of PorU and PorZ are reduced on the cell surface, resulting in increase of the 23-kDa CTD-containing PorA on the cell surface. The 23-kDa CTD-containing PorA activates the PorXY-SigP pathway and increases expression of T9SS component genes to yield increase of the T9SS operation. Further investigation is needed to reveal the role of PorA in activation of the PorXY-SigP signaling pathway.

In conclusion, we found that PorA, a T9SS CTD-containing protein, impacts the PorXY-SigP signaling pathway that is responsible for expression of the T9SS. Putative protein-encoding genes homologous to *porA* with BLAST E-value of < 0.0001 have been found only in the genus *Porphyromonas*, indicating that the presence of PorA is restricted to a very narrow range of taxa. Therefore, the cell surface protein PorA may be a good target for pharmacotherapeutically controlling the periodontal pathogen *P. gingivalis*. This study provides a new insight into the signaling pathway that regulates the T9SS.

## Methods

### Bacterial strains and plasmids

The bacterial strains and plasmids used in this study are listed in Supplemental Table S1^4,5,15,29,31,33,47–49^.

### Media and conditions for bacterial growth

Media and conditions for bacterial growth was described previously^2^. Briefly, *P. gingivalis* strains were grown anaerobically (80% N_2_, 10% CO_2_, 10% H_2_) in enriched brain–heart infusion (BHI) broth (Becton Dickinson, Franklin Lakes, NJ) or on enriched tryptic soy agar plates (Nissui, Tokyo, Japan) supplemented with 5 μg/ml hemin (Sigma, St. Louis, MO) and 0.5 μg/ml menadione (Sigma). For blood agar plates, defibrinated laked sheep blood was added to enriched tryptic soy agar at 5%. Luria–Bertani (LB) broth and LB agar plates were used for growth of *Escherichia coli* strains. Antibiotics were used at the following concentrations: ampicillin (Ap; 100 μg/ml for *E. coli*, 10 μg/ml for *P. gingivalis*), erythromycin (Em; 10 μg/ml for *P. gingivalis*), gentamicin (Gm; 50 μg/ml for *P. gingivalis*), and tetracycline (Tc; 0.7 μg/ml for *P. gingivalis*).

### Chemicals

The proteinase inhibitors Nα-*p*-tosyl-l-lysine chloromethyl ketone hydrochloride (TLCK) and leupeptin were purchased from Wako (Osaka, Japan) and Peptide Institute (Osaka, Japan), respectively. Iodoacetamide was purchased from Wako (Osaka, Japan).

### Construction of bacterial mutant strains

Construction of *P. gingivalis* mutants was described in detail in Supplemental text. Primers used in this study are listed in Supplemental Table S2.

### Construction of a complemented strain of the *ΔporA* mutant

Construction of a complemented strain of the *ΔporA* mutant was described in detail in Supplemental Text^5,47,49^.

### Electrotransformation of pTIO-*tetQ*-*porY*(S266W) in the* ΔporA* mutant

Complementation of pTIO-*tetQ*-*porY*(S266W) was described in Supplemental Text.

### Enzymatic assay

Kgp and Rgp activities were determined as previously described^30^. The determination is described in detail in Supplemental Text.

### Hemagglutinating activity

Hemagglutinating activities were determined as previously described^33^. The determination is described in detail in Supplemental Text.

### Preparation of vesicle fraction

Vesicle fraction was obtained as described previously^50^. The determination is described in detail in Supplemental Text.

### Antibodies

His_6_-tagged recombinant proteins of PorA, PorV, SigP, PorL, PorM, and PorN were overexpressed in *E. coli* BL21(DE3) carrying an expression plasmid. The expression plasmid was constructed as follows. DNA fragments of *porA*, *porV*, *sigP*, *porL*, *porM*, and *porN* gene were PCR amplified with primer sets PorA-15bF/PorA-15bR, PorV-32bF/PorV-32bR, SigP-15bF/SigP-15bR, PorL-15bF/PorL-15bR, PorM-22bF/PorM-22bR, and PorN-22bF/PorN-22bR, respectively, using *P. gingivalis* ATCC 33277 chromosomal DNA as a template. The amplified DNA of *porA* was digested with NdeI plus BglII, the amplified DNA of *sigP* and *porL* was digested with NdeI plus BamHI, and the resulting DNA fragments were inserted into the NdeI-BamHI site of pET-15b (Novagen). The amplified DNA of *porM* was digested with BamHI plus XhoI and then inserted into the BamHI-XhoI site of pET-22b(+) (Novagen). The amplified DNA of *porN* was digested with SalI plus XhoI and then inserted into the SalI-XhoI site of pET-22b(+). The amplified DNA of *porV* was digested with EcoRV plus XhoI and then inserted into the EcoRV-XhoI site of pET-32b(+) (Novagen). *E coli* BL21 (DE3) harboring the resulting plasmids were grown on LB broth at 30 °C, and PorA, PorV, SigP, PorL, PorM, and PorN were induced with 0.1 mM isopropyl-β-d-thiogalactopyranoside. His_6_-tagged recombinant proteins were purified by using a HiTrap Chelating HP column (GE Healthcare Life Sciences). To raise antiserum against PorA, SigP, PorV, PorL, PorM, and PorN, rabbits were immunized with PorA-His_6_ and guinea pigs were immunized with the SigP-His_6_ by EveBioscience Co., Ltd. (Wakayama, Japan). PorV, PorL, PorM, and PorN-His_6_ protein were mixed with TiterMax Gold (TiterMax), and the mixtures were injected into mice (BALB/c) subcutaneously, resulting in α-PorV, α-PorL, α-PorM, α-PorN antiserum. An α-Kgp rabbit polyclonal antibody^51^, an α-PorK rabbit polyclonal antibody^5^, an α-Rgp mouse polyclonal antibody^51^, an α-PorU mouse polyclonal antibody^52^, an α-Hbp35 rabbit polyclonal antibody^53^, an α-Mfa1 rabbit polyclonal antibody^54^, an α-PorX rabbit polyclonal antibody^29^, and an α-PorY rabbit polyclonal antibody^29^ were used to detect Kgp, PorK, Rgp, PorU, Hbp35, Mfa1, PorX, and PorY, respectively. A monoclonal antibody 1B5 (mAb 1B5), which recognizes the anionic polysaccharide of A-LPS, was kindly provided by Prof. M. A. Curtis^55^. An α-Halo Tag rabbit polyclonal antibody was purchased from Promega.

### Gel electrophoresis and immunoblot analysis

SDS-PAGE and immunoblot analysis were performed as previously described^15,17^.

### Dot blot analysis

Dot blot analysis was performed as previously described^15^.

### Biotinylation of surface proteins of *P. gingivalis*

Biotinylation of surface proteins in *P. gingivalis* cells was performed as previously described^56^. The procedure is described in detail in Supplemental Text.

### Immunoprecipitation with α-PorA antibody

Immunoprecipitation assay were described in detail in Supplemental Text.

### MS analysis and database search for protein identification

Proteins were identified by peptide mass fingerprinting (PMF) after in-gel tryptic digestion as previously described^5^.

### Whole genome sequencing of the *ΔporA* pseudo-revertants

*Porphyromonas gingivalis ΔporA* pseudo-revertants were grown on the enriched tryptic soy plates. Chromosomal DNA was extracted from the *ΔporA* pseudo-revertants using the MasterPure DNA purification kit for Blood Version II (Epicentre) and concentrated using ethanol precipitation then suspended in Tris–EDTA buffer pH8.0. Whole genome sequencing was performed at the Nagasaki University Genome Research Facility using the Ilumina HiSeq 2500 Sequencer (Illumina, San Diego, Calif). The sequence data were analyzed by using a standard pipeline. To identify small indels and single-nucleotide variants, sequence reads were aligned against *P. gingivalis* ATCC 33277 genome (AP009380)^57^ modified with the insertion of *ermF* to *porA*. We also used information of the *P. gingivalis* W83 genome (GenBank under accession number AE015924)^58^.

### Quantification of gene expression by quantitative RT (qRT)-PCR

qRT-PCR analysis was performed as previously described^5^. Primers used in this study are listed in Supplemental Table S2.

### Purification of Halo-CTD(PorA) chimera protein with DTBP-mediated crosslinking

Purification of Halo-CTD(PorA) chimera protein with DTBP-mediated crosslinking was described in Supplemental Text^59^.

### Purification of His-PorA(Q28-K246)

Purification od His-PorA(Q28-K246) was described in Supplemental Text.

### Limited proteolysis

Limited proteolysis was performed as previously described^60^. Purified His-PorA(Q28-K246) solution was mixed with trypsin at the protease/protein ratio of 1/100 (w/w) for 90 min at 27 °C. The products were analyzed by SDS-PAGE and MALDI-TOF Mass Spectrometry using sinapinic acid or α-Cyano-4-hydroxycinnamic acid as matrix reagents.

### Purification of PorA-N and Se-Met PorA-N

Purification procedure of PorA-N and Se-Met PorA-N was described in Supplemental Text.

### Crystallization, data collection and structure determination

Procedures of crystallization, data collection and structure determination were described in detail in Supplemental Text^61–64^. Structural refinement statistics are summarized in Supplemental Table S3.

### Data deposition

The atomic coordinates have been deposited in Protein Data Bank, http://www.pdb.org (PDB ID code 6KJK).

## Supplementary information

Supplementary Information.

## Data Availability

The atomic coordinate of the crystal structure has been deposited into the Protein Data Bank (PorA: PDB ID 6KJK). Additional raw data that support the findings of this study are available from the corresponding author upon reasonable request.
