# Supplementary material for: PorA, a conserved C-terminal domain-containing protein, impacts the PorXY-SigP signaling of the type IX secretion system
Source: Sci Rep. 2020 Dec 3;10:21109. doi: 10.1038/s41598-020-77987-y (PMC7712824; doi:10.1038/s41598-020-77987-y)
Supplement: Supplementary file 1 — Supplementary Information. [file 41598_2020_77987_MOESM1_ESM.pdf]

Supplementary Information for:

PorA, a conserved C-terminal domain-containing protein, impacts the PorXY-SigP signaling of the type IX secretion system

Hideharu Yukitake[1], Mikio Shoji[1], Keiko Sato[1], Yusuke Handa[2], Mariko Naito[1], Katsumi Imada[2], Koji Nakayama[1].

1. Department of Microbiology and Oral Infection, Graduate School of Biomedical Sciences, Nagasaki University, Nagasaki, Nagasaki 852-8588, Japan.
2. Department of Macromolecular Science, Graduate School of Science, Osaka University, Toyonaka, Osaka 560-0043, Japan

Correspondence and requests for materials should be addressed to M.S. (m-shoji@nagasaki-u.ac.jp)

Supporting Materials

Fig. S1. Colony pigmentation of *P. gingivalis* mutant strains. (A) Wild type, *ΔporA*, *ΔPGN_0654*, and *ΔPGN_1770* mutants were grown on the blood agar plate for 3 days. (B) Wild type, *ΔporA ΔporY*, and *ΔporA ΔporY*/*porY*(S266W) mutants were grown on the blood agar plate for 5 days. This pigmentation data is related to Figs. S5 and S6.

Fig. S2. Presence of PorK, PorL, and PorM proteins in the *ΔporA* mutant. Cell lysates of *P. gingivalis ΔporA*, *porK*, *porL*, and *porM* mutants were subjected to SDS-PAGE, followed by immunoblot analyses with α-PorK, α-PorL, and α-PoM. Exposure time in the experiments in this figure was much longer than that in the experiments in Fig. 2B. Black, green, and red arrows indicate the PorK, PorL, and PorM proteins, respectively. CBB: Coomassie Brilliant Blue staining.

Fig. S3. qRT-PCR analysis of the *porX* mRNA expression in the *ΔsigP* mutant. Expression of the *porX* gene in the wild type and the *ΔsigP* mutant. RNA samples of the strains were subjected to qRT-PCR analysis. The mean expression of *porX* in the wild type was regarded as 1.

Fig. S4. Plasmid-mediated complementation in the *ΔporA*, *ΔporY*, *ΔporX*, and *ΔsigP* mutants. (A) Cell lysates of *P. gingivalis* wild type, *ΔporA*, *ΔporA*/*porA*$^+$, *ΔporY*, *ΔporY*/*porY*$^+$, *ΔporX*, *ΔporX/porX*$^+$, *ΔsigP*, and *ΔsigP*/*sigP*$^+$ strains were subjected to SDS-PAGE, followed by immunoblot analysis with α-PorY. Full-length blot of Fig. S4A is presented in Fig. S12. (B) Expression of the *porY* gene in the wild type, *ΔporA*, *ΔporA*/*porA*$^+$, *ΔporY*, *ΔporY*/*porY*$^+$, *ΔporX*, *ΔporX*/*porX*$^+$, *ΔsigP*, and *ΔsigP*/*sigP*$^+$ strains. RNA samples of the strains were subjected to qRT-PCR analysis. The mean expression of *porY* in the wild type was regarded as 1.

Fig. S5. Immunoblot analysis of the *ΔporA ΔporY* mutant and the *ΔporA ΔporY*/*porY*(S266W) strain. Cell lysates of *P. gingivalis* wild type, *ΔporA*::*cepA*, *ΔporY*::*ermF*, *ΔporA ΔporY*, *ΔporA/porY*(S266W), and *ΔporA ΔporY*/*porY*(S266W) strains were subjected to SDS-PAGE, followed by immunoblot analysis with α-PorA. α-PorK, α-PorX, α-PorY, and α-SigP. CBB: Coomassie Brilliant Blue staining. The PorK, PorX, PorY, and SigP proteins are indicated by black, red, blue, and green arrows.

Fig. S6. Kgp and Rgp activities of the *ΔporA ΔporY* mutant and the *ΔporA ΔporY*/*porY*(S266W) strain.

Kgp and Rgp activities in whole cell cultures of *P. gingivalis* wild type, *ΔporA*::*ermF*, *ΔporA*/*porA*$^+$, *ΔporA*::*cepA*, *ΔporY*::*ermF*, *ΔporA ΔporY*, *ΔporA*/*porY*(S266W), and *ΔporA ΔporY*/*porY*(S266W) strains were measured.

Fig. S7. Dot blot and immunoblot analyses of various T9SS-related mutants. (A) Dot blot analyses of the wild type, *ΔporA*, *porK*, *ΔporX*, *ΔporY*, and *ΔsigP* strains with α-Hbp35 and α-PorA. (B) Immunoblot analyses with α-Hbp35 and α-PorA.

Fig. S8. Interaction between PorA and PorV. (A) Immunoblot analysis with α-PorA and α-PorV. The α-PorA-immunoprecipitated proteins from the membrane fractions of *P. gingivalis* cells treated without DTBP were separated by SDS-PAGE, followed by immunoblot analyses using α-PorA and α-PorV. CBB: Coomassie Brilliant Blue staining. (B) The same samples as (A) were subjected to Native PAGE, followed by immunoblot analyses using α-PorA and α-PorV. CBB: Coomassie Brilliant Blue staining.

Fig. S9. Interaction of PorV with CTDs of various T9SS cargo proteins. (A) Alignment of various CTDs used for Halo tag fusion proteins. The C-terminal ~80

amino acids of Hbp35, Kgp, Mfa5, PPAD, and RgpB were aligned using the ClustalW. '*' indicates positions which have a single, fully conserved residue. ':' indicates that one of the following 'strong' groups is fully conserved. '.' indicates that one of the following 'weaker' groups is fully conserved. (B) Halo tag fusion proteins were purified by Halo tag resin, followed by immunoblot analyses with α-PorV and α-Halo tag. The black arrows indicate the PorV protein.

Fig. S10. Expression of T9SS components in T9SS component-deficient mutants.

(A) Expression of the *porK*, *porL*, *porM*, *porT*, *porV*, and *sov* genes in the wild type, *porK, porM, porT, porV,* and *sov* strains. RNA samples of the strains were subjected to qRT-PCR analysis. The mean expression of each gene in the wild type was regarded as 1. (B) Production of the PorK, PorM, and PorV proteins in the wild type, *porK*, *porM*, *porT*, *porV*, and *sov* strains. Cell lysates of the strains were subjected to SDS-PAGE, followed by immunoblot analyses with α-PorK. α-PorM, and α-PorV. Full-length blot of Fig. S10B is presented in Fig. S13.

Fig. S11. Full-length blots/gels of Figs. 1E and 1F are presented.

Fig. S12. Full-length blot of Fig. S4A is presented.

Fig. S13. Full-length blots of Fig. S10B are presented.

Table S1. Bacterial strains and plasmids used in this study.

Table S2. Primers used in this study.

Table S3. X-ray data collection and refinement statistics.

Supplemental Text

Construction of *P. gingivalis* mutant strains

DNA regions upstream and downstream of the *porA*, PGN_1770, and PGN_0654 genes were PCR amplified from the chromosomal DNA of *P. gingivalis* ATCC 33277 using pairs of primers (PGN gene number-U-F plus PGN gene number-U-R and PGN gene number-D-F plus gene number -D-R), respectively, where *U* indicates upstream, *F* indicates forward, *D* indicates downstream, and *R* indicates reverse. Primers used in this study are listed in Supplemental Table S2. The amplified DNA upstream of each gene was double digested with KpnI plus a corresponding restriction enzyme (BamHI or BglII). The amplified DNA downstream of each gene was digested with NotI plus a corresponding restriction enzyme (BamHI or BglII). Both digested DNAs were inserted together into the KpnI-NotI site of pBluescript II SK(+) to yield pKD1301, pKD1308, and pKD1309. The 1.1-kb BamHI *ermF* DNA fragment was inserted into the BamHI or BglII site of the resulting plasmids to yield plasmids pKD1302, pKD1310, and pKD1311 for mutagenesis. The 1.17-kb BamHI *cepA* DNA fragment was inserted into the BglII site of the pKD1301 to yield plasmid pKD1303 for mutagenesis. The pKD1302 was digested with BssHII and the pKD1310, pKD1311, and pKD1303 were digested with KpnI plus NotI, and these linearized DNA fragments were introduced into

P. gingivalis ATCC 33277 by electroporation to generate Em^r transformant (KDP1041, KDP1049, and KDP1050) or Ap^r transformant (KDP1045).

To create a *kgp*::*cat rgpA*::*cepA rgpB*::*tetQ ΔporA*::*ermF* mutant (KDP1040), BssHII-digested pKD1302 was introduced into KDP981 (*kgp*::*cat rgpA*::*cepA rgpB*::*tetQ*) by electroporation followed by selection on blood agar plates containing Em (10 μg/ml).

To create a *porU*::*ermF ΔporR::tetQ* mutant (KDP1059), the pKD894 were linearized with NotI and introduced into *porU*::*ermF* (KDP360) by electroporation followed by selection on blood agar plates containing Tc (0.7 μg/ml).

Construction of a complemented strain of the *ΔporA* mutant

The promoter region of *Porphyromonas gulae catalase* gene (accession no. AB083039 in GenBank/EMBL/DDBJ databases) was PCR amplified from *P. gulae* VPB3492 chromosomal DNA using the pair of primers PRO-U and PRO-R-B, digested with KpnI plus BamHI, and inserted into the KpnI-BamHI site of pBSSK to yield pKD1304. The entire *porA* gene containing its downstream region was PCR amplified from *P. gingivalis* ATCC 33277 chromosomal DNA using the pair of primers N0123-coF-B/N0123-coR-(NotI), the amplified DNA was cloned into the pGEM-T Easy vector

(Promega) to yield pKD1305. pKD1305 DNA was digested with BglII plus NotI (restriction site within the pGEM-T Easy vector), and then ligated with the larger BamHI-NotI fragment of pKD1304 to construct pKD1306. The smaller KpnI-NotI fragment of pKD1306 was then ligated with the larger KpnI-NotI fragment of pTCB to construct pKD1307. After mating of *Escherichia coli* S17-1[47] containing pKD1307 with the *P. gingivalis ΔporA* mutant KDP1041 or KDP1045, an Em^r Tc^r transconjugant (KDP1042) or an Ap^r Tc^r transconjugant (KDP1051) were obtained.

Electrotransformation of pTIO-*tetQ-porY*(S266W) in the *ΔporA* mutant

The entire *porY* containing its promoter was PCR-amplified from chromosomal DNA of a pseudo-revertant containing *porY*(S266W) of the *ΔporA* mutant using the pair of primers porY-U-F-P and porY-D-R-N, digested with PstI plus NotI, and then inserted into the PstI–NotI site of the *E. coli-Bacteroides* shuttle vector plasmid pTIO-*tetQ* to yield pTIO-*tetQ-porY*(S266W). The plasmids pTIO-*tetQ* and pTIO-*tetQ-porY*(S266W) were introduced into the *ΔporA* mutants KDP1041 and KDP1045 by electroporation, resulting in strains KDP1043 and KDP1044, and strains KDP1046 and KDP1047, respectively. To construct KDP1048, a 2.7-kb fragment containing the *ΔporX*::*ermF* gene region was PCR amplified from the KDP363 chromosomal DNA using the pair of

primers N1019-U-F3/N1019-D-R2. Amplified DNA was introduced into KDP1047 by transformation, resulting in KDP1048.

Construction of *ΔporA ΔporY* double mutant strains

To construct *ΔporA*::*cepA ΔporY*::*ermF* mutant (KDP1060) and *ΔporA*::*cepA ΔporY*::*ermF* /pTIO-*tetQ*-p-*porY*(S266W) mutant (KDP1061), a 2.2-kb fragment containing the *ΔporY*::*ermF* gene region was PCR amplified from the *ΔporY*::*ermF* mutant (KDP364) chromosomal DNA using the pair of primers PGN2001-U-F-650/ PGN2001-D-R-502. Amplified DNA was introduced into the *ΔporA*::*cepA* mutant (KDP1045) and *ΔporA*::*cepA*/pTIO-*tetQ*-p-*porY*(S266W) mutant (KDP1047) by electroporation, resulting in KDP1060 and KDP1061, respectively.

Dot blot analysis

P. gingivalis cells that had fully grown in enriched BHI medium were harvested, washed once with PBS and suspended in PBS. The washed cells were adjusted to an OD_{595} of 0.5. A 3 µl aliquot of the adjusted cells was blotted directly onto a nylon membrane (Biodyne Plus) and left to dry.

Biotinylation of surface proteins of *P. gingivalis*

P. gingivalis cells that had fully grown in enriched BHI medium were harvested by centrifugation, washed once with ice-cold PBS, suspended in PBS containing 1 mg/ml biotinamidocaproate *N*-hydroxysuccinimide ester (Calbiochem-Novabiochem) and mixed gently for 30 min at 37 °C[56]. The labelled cells were washed twice with ice-cold PBS and lysed with 1% DDM (vol/vol) and then immunoprecipitated by the use of Protein G agarose beads with 5 μl of α-PorA polyclonal antibody. The immunoprecipitated samples were subjected to SDS-PAGE. Biotinylated proteins on the gel were transferred to a PVDF membrane and detected with peroxidase-conjugated streptavidin (Chemicon).

Immunoprecipitation with α-PorA antibody

Cells were grown in BHI broth (200 ml) until late exponential phase (overnight culture). Cells were harvested by centrifugation at 10,000 × *g* for 20 min and washed with HEPES buffer (10 mM HEPES, pH 8.0, 0.15 M NaCl) two times and concentrated eight-fold by suspension in 25 ml of 10 mM HEPES pH8.0. Cells were cross-linked using 0.25 mM dimethyl 3,3'-dithiobispropionimidate (DTBP) (easily reversed with dithiothreitol (DTT)) for 1 h at room temperature. The reactions were quenched for 10

min with 1.25 ml (50 mM final concentration) of 1 M Tris-HCl (pH 8.0). Cells were centrifuged at 10,000 × *g* for 10 min and suspended in 20 ml of Buffer A (10 mM HEPES pH 8.0 with 1 mM TLCK, 1 mM Leupeptin) and then mixed with 25 µg/ml DNase I and 25 µg/ml RNase A. After gentle shaking for 15 min at 4 °C, the suspension was disrupted by three passes at 100 MPa in a French pressure cell press. Remaining intact bacteria cells were removed by centrifugation at 2,400 × *g* for 10 min. Supernatant was centrifuged at 100,000 × *g* for 60 min at 4 °C. The pellets were suspended in Buffer A and then mixed with 1% DDM (vol/vol). After gentle shaking for 30 min at RT, the suspension was centrifuged at 8,000 × *g* for 10 min at RT. The supernatant was stored at 4 °C as the membrane fraction. The membrane fraction was immunoprecipitated by use of Protein G agarose beads with 5 µl of α-PorA polyclonal antibody. Before the immunoprecipitation analysis, the α-PorA polyclonal antibody was cross-linked to the Protein G beads by dimethyl pimelimidate dihydrochloride to prevent co-elution of the antibody with the target protein. In Figs. 4A and S8A, the resulting precipitate was dissolved with the same volume of the sample buffer and loaded on an SDS (12%) gel. In Fig. S8B, the resulting precipitate was dissolved with the elution buffer (0.1 M glycine-HCl, pH 2.7), and then supplemented with

neutralization buffer (1 M Tris-HCl, pH 9.0) and loaded on a Native gel. Immunoblot analysis was performed with α-PorA and α-PorV.

Hemagglutinating activity

Overnight cultures of *P. gingivalis* strains grown in enriched BHI medium were centrifuged, washed once with PBS, and suspended in PBS at an optical density of 0.5 at 595 nm. The bacterial suspensions were then diluted in a twofold series with PBS. A 100-μl aliquot of each suspension was mixed with an equal volume of defibrinated sheep erythrocyte suspension (1% in PBS) and incubated in a round-bottom microtiter plate at room temperature (RT) for 3 h.

Enzymatic assay

When we measured Kgp and Rgp activities in cells or in culture supernatants in Figs. 1C and 2D, cultures of *P. gingivalis* strains at OD_{600} of 1.0 were centrifuged to separate the culture supernatants and cells. Precipitated cells were resuspended with an original volume of phosphate-buffered saline (PBS). Each volume (2 μl for Kgp and 5 μl for Rgp) of bacterial cell suspensions and of culture supernatants were added to the reaction mixture (1.0 ml) containing 5 mM cysteine, 20 mM sodium phosphate buffer, pH 7.5, and 10 mM benzyloxycarbonyl-L-histidyl-L-glutamyl-L-lysine 4-methylcoumaryl-7-amide (Peptide Institute,Osaka, Japan) (for Kgp) or benzyloxycarbonyl-L-phenylalanyl-

L-arginine 4-methylcoumaryl-7-amide (Peptide Institute) (for Rgp). After 10 min incubation at 40 ºC, the reaction was terminated by adding 100 mM sodium acetate buffer, pH 5.0, containing 10 mM iodoacetic acid (1.0 ml). The released 7-amino-4-methyl-coumarine was measured at 465 nm (excitation at 365 nm) by fluorescence spectrophotometer Beckman Coulter DTX 800 (Brea, CA). Enzymatic activities are expressed as ΔE/min/µl. Whole cell cultures without separation to cells and culture supernatants were used in the experiments shown in Fig. S6.

Preparation of the outer membrane vesicle fraction

P. gingivalis strains were mono- or co-cultured in 60 ml of enriched BHI medium at 37 °C for 48 h. *P. gingivalis* cells were harvested by centrifugation at 6,000 × *g* for 20 min at 4 °C. The supernatants were ultracentrifuged at 100,000 × *g* for 60 min at 4 °C. The pellets were washed once with PBS, and then dissolved in PBS containing 0.1 mM TLCK and 0.1 mM Leupeptin.

MS analysis

A gel plug containing proteins was subjected to the following procedures: washing with 50% (v/v) acetonitrile, washing with 100% acetonitrile, reduction with 10 mM DTT, alkylation with 55 mM iodoacetamide, washing/dehydration with 50% (v/v) acetonitrile, and digestion for 10 h with 10 µg/ml trypsin. The resulting peptides were

extracted from the gel plug with 0.1% (v/v) trifluoroacetic acid/50% (v/v) acetonitrile. Digests were spotted on a MALDI target using α-cyano-4-hydroxycinnamic acid as a matrix. Spectra were acquired on a 4800 MALDI TOF/TOF analyzer (Applied Biosystems). Data analysis and MS database searching were performed using GPS ExplorerTM and MASCOT software.

qRT-PCR analysis

Total RNA was isolated from *P. gingivalis* cells grown to mid-exponential phase (OD_{600} of ~1.0) by using a TRIzol reagent (Invitrogen, Carlsbad, CA, USA) and RNeasy Mini Kit (Qiagen Science). DNA was removed with RNase-Free DNase. cDNA was generated with SuperScript® VILO cDNA Synthesis Kit (Invitrogen). Real-time quantitative PCR (qRT-PCR) was performed using Brilliant III Ultra-Fast SYBR Green qRT-PCR Master Mix (Agilent Technologies, Santa Clara, CA) with the Mx3005P™ Real-Time PCR System (Agilent Technologies) according to the manufacturer's instruction. Primers for qRT-PCR were designed using Primer3 program and are listed in Table S2. qRT-PCR conditions were as follows: 1 cycle at 95 °C for 10 min, and 35 cycles of 95 °C for 30 s, and 60 °C for 1 min. At each cycle, accumulation of PCR products was detected by the reporter dye from the dsDNA-binding SYBR Green. To

confirm that a single PCR product was amplified, after the PCR, a dissociation curve (melting curve) was constructed in the range of 55 °C to 95 °C. All data were analyzed using Mx3005P software. The expression level of each targeted gene was normalized to that of the 16S rRNA gene, which was used as a reference. All PCR reactions were carried out in triplicate. The efficiency of primers binding was determined by linear regression by plotting the cycle threshold (C_T) value versus the log of the cDNA dilution. Relative quantification of transcript was determined using the comparative C_T method ($2^{-\Delta\Delta CT}$) calibrated to 16S rRNA. qRT-PCR experiments were performed multiple times independently with comparable results.

Construction of Halo-CTD chimera proteins

The signal sequence region coding for amino acids (M1-A19) of the *kgp* gene containing its promoter region was PCR amplified from *P. gingivalis* ATCC 33277 chromosomal DNA using the pair of primers kgp-U400-F-KpnI/kgp-sigR-SalI, digested with KpnI plus SalI, and inserted into the KpnI-SalI site of pBSSK to yield pKD1312. The entire HaloTag gene region was PCR amplified from pFC15A containing Halo-tag (Promega) using the pair of primers Halo-F-SalI/ Halo-stopR-BglII or Halo-R-BglII. The amplified DNAs were cloned into the pGEM-T Easy vector (Promega) to yield

pKD1313 and pKD1314. The sequence region coding for CTD of each T9SS cargo protein was PCR amplified from *P. gingivalis* ATCC 33277 chromosomal DNA using the pair of primers porA-CTD84-F-BglII/porA-CTD-R-NotI, rgpB-CTD73-F-BglII/rgpB-CTD-R-NotI, ppad-CTD84-F-BglII/ppad-CTD-R-NotI, mfa5-CTD84-F-BglII/mfa5-CTD-R-NotI, hbp35-CTD67-F-BglII/hbp35-CTD-R-NotI, kgp-CTD72-F-BglII/kgp-CTD-R-NotI. The each amplified DNA was cloned into the pGEM-T Easy vector (Promega) to yield pKD1315, pKD1316, pKD1317, pKD1318, pKD1319, and pKD1320. The above plasmid was digested with BglII plus NotI, and then ligated with the larger BglII-NotI fragment of pKD1314 to construct pKD1321, pKD1322, pKD1323, pKD1324, pKD1325 and pKD1326. The smaller SalI-NotI fragments of these plasmids were then ligated with the larger SalI-NotI fragment of pKD1312 to construct pKD1327, pKD1328, pKD1329, pKD1330, pKD1331, and pKD1332, respectively. The smaller SalI plus NotI (restriction site within the pGEM-T Easy vector) fragment of pKD1313 plasmid was then ligated with the larger SalI-NotI fragment of pKD1312 to construct pKD1333. The smaller KpnI-NotI fragments of pKD1327, pKD1328, pKD1329, pKD1330, pKD1331, pKD1332, and pKD1333 were then ligated with the KpnI-NotI site of pTCB plasmid to construct pKD1334, pKD1335, pKD1336, pKD1337, pKD1338, pKD1339, and pKD1340, respectively. After mating

of *Escherichia coli* S17-1[47] containing each pKD1334, pKD1335, pKD1336, pKD1337, pKD1338, pKD1339, and pKD1340 with the *P. gingivalis porN* mutant KDP358, Em^r Tc^r transconjugants (KDP1052, KDP1053, KDP1054, KDP1055, KDP1056, KDP1057, and KDP1058) were obtained, respectively.

Isolation of Halo-CTD chimera protein fraction with DTBP-mediated crosslinking from partially purified samples

Isolation of Halo-CTD(PorA) chimera protein fraction with DTBP-mediated crosslinking was performed as described previously[59]. Partially purified samples (PP) were prepared as follows. Cells were grown in BHI broth (200 ml) until late exponential phase (overnight culture). Cells were harvested by centrifugation at 10,000 × *g* for 20 min and washed twice with HEPES buffer (10 mM HEPES pH 8.0, 0.15 M NaCl) and concentrated eight-folds by suspension in 25 ml of HEPES buffer. Samples were cross-linked using 0.25 mM DTBP (easily reversed with DTT) for 1 h at room temperature. The reactions were quenched for 10 min with 1.25 ml (final 50 mM) of 1 M Tris-HCl (pH 8.0). Samples were centrifuged at 10,000 × *g* for 10 min and suspended in 40 ml ice-cold solution (step-1: 30 mM Tris-HCl pH 7.4, protease inhibitor cocktail (PIC): 1 mM TLCK, 1 mM EDTA pH 8.0, 500 mM sucrose) and incubated for 10 min on ice.

Samples were centrifuged at 8,000 × *g*, 4 °C for 25 min. Pellets were resuspended in 40 ml ice-cold solution (step-2: 5 mM $MgCl_2$, PIC) and gently mixing for 10 min. Samples were centrifuged at 8,000 × *g*, 4 °C for 25 min and resulting supernatants were concentrated by Amicon Ultra-15, 10K (Merck KGaA). One ml of concentrated supernatants was mixed with 12.5 µl of settled HaloLink Resin and incubated for 1 h at room temperature. After centrifugation at 4 °C for 5 min, the supernatants were removed and the resin was washed three times with 1 ml of HaloTag purification buffer (1×TBS [prepared from 10×TBS; 1 M Tris, 1.5 M NaCl, pH 7.6] and 0.05% IGEPAL-CA630). After centrifugation, the resin was dissolved with 50 µl of 2×SDS sample buffer containing DTT and heat denatured at 85 °C for 3 min.

Purification of PorA-N

His-PorA (Q28-K246) was expressed and purified with Ni-NTA affinity chromatography as described above. The His-tag and CTD of His-PorA (Q28-K246) were removed by the addition of trypsin (Sigma) for 1 h at 27 °C, and the reaction was terminated by addition of TLCK. The reactant was dialyzed against 10 mM Tris-HCl pH 8.0 at 4 °C overnight and loaded onto an anion exchange column (a HiTrap HP column (GE Healthcare)). Proteins were eluted with a linear gradient of NaCl (0-300

mM) and peak fractions containing PorA-N were further purified by size exclusion chromatography with a High Load 26/60 Superdex 200 column. The peak fractions were concentrated by ultrafiltration to 15.0 mg/ml, using an Amicon Ultra device (Merck Millipore), and used for crystallization.

Purification of His-PorA (Q28-K246)

E. coli BL21 (DE3) (Novagen) cells carrying pET15b-PorA (Q28-K246) were grown in LB medium (1% (w/v) Bacto tryptone, 0.5% (w/v) yeast extract, 0.5% (w/v) NaCl) containing 50 μg/ml Ap at 30 °C until the cell density had reached an OD_{660} of around 0.5. Isopropyl β-d-1-thiogalactopyranoside (IPTG) was added to a final concentration of 0.2 mM to induce protein expression, and the culture was continued for 18 h at 18 °C. Cells were harvested by centrifugation and suspended in Buffer A (50 mM Tris-HCl (pH 8.0), 500 mM NaCl) containing protease inhibitor cocktail (Sigma). The cells were disrupted by sonication, and the soluble fraction isolated by ultracentrifugation was loaded onto a Ni-NTA affinity column (a HisTrap column (GE healthcare)). Proteins were eluted with a linear gradient of imidazole (0-350 mM). Peak fractions containing His-PorA (Q28-K246) were corrected and further purified with a High Load 26/60 Superdex 200 column (GE healthcare).

Purification of Se-Met PorA-N

E. coli BL21 (DE3) cells carrying pET15b-PorA (Q28-K246) were inoculated in LB medium containing 50 µg/ml Ap at 30 °C for 20 h. Cells were harvested by centrifugation, washed and suspended in sterilized 100 ml of 0.9% NaCl solution. The cell suspension was mixed with 400 ml of Se-Met medium (1 g/l NH_4Cl, 3 g/l KH_2PO_4, 7.7 g/l Na_2HPO_4, 20 g/l glucose, 0.3 g/l $MgSO_4$, 10 mg/l $Fe_2(SO_4)_3$, 10 mg/l thiamine, 50 mg/l L-selenomethionine) containing 50 µg/ml Ap and cultured at 30 °C until the cell density had reached an OD_{660} of around 0.5. Then IPTG was added to a final concentration of 0.2 mM, and the culture was continued for 18 h at 30 °C. Se-Met PorA-N was purified in the same way as native PorA-N.

Crystallization

Initial screening was performed using the sitting-drop vapor-diffusion technique with commercially available screening kits Wizard I and II, Cryo I and II (Emerald BioSystems) and Crystal Screen I and II (Hampton Research) at 293 K. Each drop was prepared by mixing 0.5 µl of protein solution (12 or 15 mg/ml, 10 mM Tris pH 8.0, 0.1 M NaCl) with 0.5 µl of reservoir solution and equilibrated to 70 µl of reservoir solution.

The best crystals were obtained from the drop prepared by mixing 0.5 µl of 12 mg/ml protein solution with 0.5 µl of reservoir solution containing 26% PEG8000 and 0.1 M $(NH_4)_2SO_4$ at 293 K. Rod crystals grew to typical dimensions of 0.1 x 0.1 x 0.3 mm. The space group of the crystals was *C*2 with unit cell dimensions *a* = 67.3, *b* = 43.2, *c* = 48.6 Å and beta = 108.5°. Se-Met derivative crystals were grown in similar condition (0.5 µl of 18 mg/ml protein solution with 0.5 µl of reservoir solution containing 22% PEG8000 and 0.1 M $(NH_4)_2SO_4$ at 293 K) as the native crystals. The crystals belong to the space group of *C*2 with unit cell dimensions *a* = 67.6, *b* = 43.3, *c* = 48.8 Å and beta = 108.7°.

Data collection and structure determination

X-ray diffraction data were collected at beamlines BL41XU in SPring-8 (Harima, Japan) with the approval of the Japan Synchrotron Radiation Research Institute (JASRI) (Proposal No.2014B1478, 2016A2539 and 2016B2539). Crystals were soaked in a cryo-protectant solution containing 10% (v/v) 2-methyl-2,4-pentanediol and 90% (v/v) of the reservoir solution for several seconds and were immediately transferred into liquid nitrogen for freezing. The diffraction data were measured under nitrogen gas flow at 100K. The anomalous diffraction data of the Se-Met crystals were collected at the

wavelength of 0.979 Å. The statistics of the diffraction data are summarized in Table S3. The diffraction data were processed with MOSFLM[61] and were scaled with Aimless[62]. The experimental phase was calculated using the SAD data of the Se-Met derivative and the initial model was automatically constructed using the program Phenix[63]. The atomic model was manually modified with Coot[64] and refined to 1.3 Å with Phenix against the native crystal data that showed the best resolution limit. The final refinement R factor and the free R factor were converged to 17.8% and 19.7%, respectively. The Ramachandran plot indicated that 99.3% and 0.7% residues were in the most favored and allowed region, respectively. Structural refinement statistics are summarized in Supplemental Table S3.

Ethics statement

Animal care and experimental procedures were performed in accordance with the Guidelines for Animal Experimentation of Nagasaki University with approval from the Institutional Animal Care and Use Committee (approval number 1205010983).

Supplemental Table S1. Bacterial strains and plasmids used in this study.

Strain or plasmid	Description	Reference or source
E. coli strain		
XL-1 blue	Host strain for general cloning	Stratagene
BL21(DE3)	Host strain for expression vectors	Nippongene
S17-1	RP4-2-Tc::Mu *aph*::Tn7 *recA*, Sm^r	(47)
P. gingivalis strain		
ATCC 33277	Wild type	
KDP117	*porT1*::*kan ermF*, Em^r	(4)
KDP220	*wbpB*::*ermF*, Em^r	(33)
KDP221	*ΔporR*::*tetQ*, Tc^r	(33)
KDP355	*porK*::*ermF*, Em^r	(5)
KDP357	*porM*::*ermF*, Em^r	(5)
KDP358	*porN*::*ermF*, Em^r	(5)
KDP360	*porU*::*ermF*, Em^r	(5)
KDP361	*porV*::*ermF*, Em^r	(15)
KDP363	*ΔporX*::*ermF*, Em^r	(5)
KDP364	*ΔporY*::*ermF*, Em^r	(5)
KDP365	*sov*::*ermF*, Em^r	(5)
KDP372	*ΔporX*::*ermF fimA::*[*porX*$^+$ *tetQ*], Em^r Tc^r	(5)
KDP380	*ΔporY::ermF fimA::*[*porY*+ *tetQ*], Em^r Tc^r	(5)
KDP391	*ΔsigP*::*ermF*, Em^r	(29)
KDP393	*ΔsigP*::*ermF*/pTCB-promoter-*sigP*+-myc-terminator, Em^r Tc^r	(29)
KDP981	*kgp*::*cat rgpA*2::*cep rgpB*2::*tetQ*, Cm^r Ap^r Tc^r	(48)
KDP982	*kgp*::*cat rgpA*2::*cep rgpB*2::*tetQ porK*::*ermF*, Cm^r Ap^r Tc^r Em^r	(48)
KDP1040	*kgp*::*cat rgpA*2::*cep rgpB*2::*tetQ ΔporA*::*ermF*, Cm^r Ap^r Tc^r Em^r	This study
KDP1041	*ΔporA*::*ermF*, Em^r	This study
KDP1042	*ΔporA*::*ermF*/pTCB-*porA*$^+$, Em^r Tc^r	This study

KDP1043	*ΔporA*::*ermF*/pTIO-*tetQ*, Em^{r} Tc^{r}	This study
KDP1044	*ΔporA*::*ermF*/pTIO-*tetQ*-p-*porY*(S266W), Em^{r} Tc^{r}	This study
KDP1045	*ΔporA*::*cepA*, Ap^{r}	This study
KDP1046	*ΔporA*::*cepA*/pTIO-*tetQ*, Ap^{r} Tc^{r}	This study
KDP1047	*ΔporA*::*cepA*/pTIO-*tetQ*-p-*porY*(S266W), Ap^{r} Tc^{r}	This study
KDP1048	KDP1047 *ΔporX*::*ermF*, Ap^{r} Tc^{r} Em^{r}	This study
KDP1049	*Δ*PGN_1770::*ermF*, Em^{r}	This study
KDP1050	*Δ*PGN_0654::*ermF*, Em^{r}	This study
KDP1051	*ΔporA*::*cepA*/pTCB-*porA*$^{+}$, Ap^{r} Tc^{r}	This study
KDP1052	*porN*::*ermF*/pTCB-p-ss(*kgp*)-*halo-porA* CTD (D163-K246), Em^{r} Tc^{r}	This study
KDP1053	*porN*::*ermF/*pTCB-p-ss*(kgp)-halo-rgpB* CTD (S664-K736), Em^{r} Tc^{r}	This study
KDP1054	*porN*::*ermF*/pTCB-p-ss(*kgp*)-*halo-pad* CTD (G473-K556), Em^{r} Tc^{r}	This study
KDP1055	*porN*::*ermF*/pTCB-p-ss(*kgp*)-*halo-mfa5* CTD (Y1145-N1228), Em^{r} Tc^{r}	This study
KDP1056	*porN*::*ermF*/pTCB-p-ss(*kgp*)-*halo-hbp35* CTD (A278-P344), Em^{r} Tc^{r}	This study
KDP1057	*porN*::*ermF*/pTCB-p-ss(*kgp*)-*halo-kgp* CTD (G1452-K1723), Em^{r} Tc^{r}	This study
KDP1058	*porN*::*ermF*/pTCB-p-ss(*kgp*)-*halo* stop, Em^{r} Tc^{r}	This study
KDP1059	*porU*::*ermF ΔporR::tetQ,* Em^{r} Tc^{r}	This study
KDP1060	*ΔporA*::*cepA ΔporY*::*ermF*, Ap^{r} Em^{r}	This study
KDP1061	*ΔporA*::*cepA ΔporY*::*ermF*/pTIO-*tetQ*-p-*porY*(S266W), Ap^{r} Em^{r} Tc^{r}	This study
Plasmid		
pBluescript II SK(+)	general cloninng vector	Stratagene
pFC15A	Halo tag containing vactor	Promega
pGEM-T Easy	general cloninng vector	Promega
pUC118	general cloninng vector	Takara
pTCB	*E. coli-P. gingivalis* shuttle plasmid, Ap^{r} Tc^{r}	(49)

pTIO-*tetQ*	*E. coli-P. gingivalis* shuttle plasmid, Ap^r Tc^r	(31)
pTIO-*tetQ*-p-*porY*[S266W]	pTIO-*tetQ-porY* promoter-*porY*[S266W], Ap^r Tc^r	This study
pKD894	pCR4-PGN_1236up-tetQ-PGN_1236dw, Ap^r Km^r Tc^r	(33)
pKD1301	pBSSK-*porA*-upstream and *porA*-downstream, Ap^r	This study
pKD1302	pBSSK-*porA*-upstream, *ermF* and *porA*-downstream, Ap^r	This study
pKD1303	pBSSK-*porA*-upstream, *cepA* and *porA*-downstream, Ap^r	This study
pKD1304	pBSSK-*cat* promoter, Ap^r	This study
pKD1305	pGEM-*porA*$^+$, Ap^r	This study
pKD1306	pBSSK-*cat* promoter and *porA*, Ap^r	This study
pKD1307	pTCB-*cat* promoter-*porA*$^+$, Ap^r Tc^r	This study
pKD1308	pBSSK-PGN_1770-upstream and PGN1770-downstream, Ap^r	This study
pKD1309	pBSSK-PGN_0654-upstream and PGN0654-downstream, Ap^r	This study
pKD1310	pBSSK-PGN_1770-upstream, *ermF* and PGN1770-downstream, Ap^r	This study
pKD1311	pBSSK-PGN_0654-upstream, *ermF* and PGN0654-downstream, Ap^r	This study
pKD1312	pBSSK-*kgp* promoter and signal sequence (ss), Ap^r	This study
pKD1313	pGEM-*halo*-tag stop, Ap^r	This study
pKD1314	pGEM-*halo*-tag, Ap^r	This study
pKD1315	pGEM-*porA* CTD84, Ap^r	This study
pKD1316	pGEM-*rgpB* CTD73, Ap^r	This study
pKD1317	pGEM-*pad* CTD84, Ap^r	This study
pKD1318	pGEM-*mfa5* CTD84, Ap^r	This study
pKD1319	pGEM-*hbp35* CTD67, Ap^r	This study
pKD1320	pGEM-*kgp* CTD72, Ap^r	This study
pKD1321	pGEM-*halo* and *porA* CTD84, Ap^r	This study
pKD1322	pGEM-*halo* and *rgpB* CTD73, Ap^r	This study
pKD1323	pGEM-*halo* and *pad* CTD84, Ap^r	This study

pKD1324	pGEM-*halo* and *mfa5* CTD84, Ap^r	This study
pKD1325	pGEM-*halo* and *hbp35* CTD67, Ap^r	This study
pKD1326	pGEM-*halo* and *kgp* CTD72, Ap^r	This study
pKD1327	pBSSK-*kgp* promoter-ss and *halo* and *porA* CTD84, Ap^r	This study
pKD1328	pBSSK-*kgp* promoter-ss and *halo* and *rgpB* CTD73, Ap^r	This study
pKD1329	pBSSK-*kgp* promoter-ss and *halo* and *pad* CTD84, Ap^r	This study
pKD1330	pBSSK-*kgp* promoter-ss and *halo* and *mfa5* CTD84, Ap^r	This study
pKD1331	pBSSK-*kgp* promoter-ss and *halo* and *hbp35* CTD67, Ap^r	This study
pKD1332	pBSSK-*kgp* promoter-ss and *halo* and *kgp* CTD72, Ap^r	This study
pKD1333	pBSSK-kgp promoter-ss and *halo* stop, Ap^r	This study
pKD1334	pTCB-*kgp* promoter-ss-*halo-porA* CTD84, Ap^r Tc^r	This study
pKD1335	pTCB-*kgp* promoter-ss-*halo-rgpB* CTD73, Ap^r Tc^r	This study
pKD1336	pTCB-*kgp* promoter-ss-*halo-pad* CTD84, Ap^r Tc^r	This study
pKD1337	pTCB-*kgp* promoter-ss-*halo-mfa5* CTD84, Ap^r Tc^r	This study
pKD1338	pTCB-*kgp* promoter-ss-*halo-hbp35* CTD67, Ap^r Tc^r	This study
pKD1339	pTCB-*kgp* promoter-ss-*halo-kgp* CTD72, Ap^r Tc^r	This study
pKD1340	pTCB-*kgp* promoter-ss-*halo* stop, Ap^r Tc^r	This study

Supplemental Table S2. Primers used in this study.

Primer	Sequence	Description
Expression of recombinant proteins for antiserum		
porA-15bF	catatgaaaaaaacaaaaagaaatatgagg	NdeI-*porA* (N0123)
porA-15bR	agatctttacttaatcagatacttctgaac	BglII-*porA* (N0123)
porL-15bF	catatgggtcattatagaagatacaagaac	NdeI-*porL* (N1675)
porL-15bR	ggatccttataagggtgagctgccggatga	BamHI-*porL* (N1675)
porM-22bF	ggatccatggcagtaggttctaatgggaat	BamHI-*porM* (N1674)
porM-22bR	ctcgaggttcacaattacttcaatggccggaa	XhoI-*porM* (N1674)
porN-22bF	gtcgacgaaaatacgaacaaccgctctccg	SalI-*porN* (N1673)
porN-22bR	ctcgagcttgcggcgacgaaccgagcgagt	XhoI-*porN* (N1673)
porV-32bF	gatatcctactaggtaatgcggagagcttg	EcoRV-*porV* (N0023)
porV-32bR	ctcgaggtggaacaaattgcgcaatccatc	XhoI-*porV* (N0023)
sigP-15bF	catatgagcagtttccacaagctgactgat	NdeI-*sigP*
sigP-15bR	ggatccctaagccgacatgcccatcatttt	BamHI-*sigP*
Expression of recombinant proteins for crystallization		
porA-15b28F	catatgcaagttgtgatcaaggtgggagat	NdeI-*porA* (N0123)
Mutation		
N0123-U-F	ggtacctgtcggtatacatgccggccctgc	KpnI-N0123-U
N0123-U-R	agatcttatttgccatcggattgcggattg	BglII-N0123-U
N0123-D-F	agatcttaatgggagtacggccaaagcctg	BglII-N0123-D
N0123-D-R	gcggccgcaaaagcctgtcccattccgca	NotI-N0123-D
N1770-U-F	ggtaccacccttttggtgatatagcatcg	KpnI-N1770-U
N1770-U-R	ggatccaagagcagcaccaataagtaatgc	BamHI-N1770-U
N1770-D-F	ggatccgagtaatattgtatacctgatag	BamHI-N1770-D
N1770-D-R	ctcccgtacagaagccaaggccttgatcg	(NotI)-N1770-D
N0654-U-F	ggtaccgtagctgatataaaagaagcaccc	KpnI-N0654-U
N0654-U-R	ggatcctttgtcttcaaatgattttagttg	BglII-N0654-U
N0654-D-F	ggatcccgatgaaacgaataattttattgc	BglII-N0654-D
N0654-D-R	cattgtaggttggtatgttgtaagtcat	(NotI)-N0654-D
N1019-U-F3	ttcctattgatggaaaacatcaca	N1019-U-941
N1019-D-R2	tatgatgacgattttactgcaa	N1019-D-685
PGN1676-U-F-NotI	gcggccgcgtgaactgaccggtgccaagc	NotI-N1676-U
PGN1676-D-R-KpnI	ggtaccgtgagcgcagctttgtattccagt	KpnI-N1676-D
PGN2001-U-F-650	caatgtgtggggatttcgtgct	N2001-U

PGN2001-D-R-502	gttcctctgcggtaaacggctcca	N2001-R
Complementation		
PRO-U	ggtaccttcgtcgtcaatcagcatcccag	KpnI-cat pro-F
PRO-R-B	ggatcctgttttgtctcttatttaagtta	BamHI-cat pro-R
N0123-coF-B	agatctatgaaaaaaacaaaaagaaatatg	BglII-N0123-F
N0123-coR-(NotI)	gggaaaaccactttgtgacgagcggtaagc	N0123-D
qRT-PCR		
REALpgn0274F	gagccaggttgcagtctttc	used for Fig. 2C, 2E
REALpgn0274R	gctcatgtcctcccagtagc	used for Fig. 2C, 2E
REALpgn1019F	gatcggggacagaagtacca	used for Fig. 2C, 2E, Supplemental Fig. S3
REALpgn1019R	attcgggtaggcgaagaagt	used for Fig. 2C, 2E, Supplemental Fig. S3
REALpgn0778F3	tctcggatgcgatttttacc	used forl Fig. 2E
REALpgn0778R3	ctcgaaattgaacgtgagca	used for Fig. 2E
REALpgn1676F1	gtccgcttagcagcgaatac	used for Fig. 2C, 2E
REALpgn1676R1	gatctgtccttcaggcaagc	used for Fig. 2C, 2E
REALpgn1675F1	cgtcgcagcacaaagaaata	used for Fig. 2C
REALpgn1675R1	aaagcatctcattgcccatc	used for Fig. 2C
REALpgn1674F1	agtaggcagcgaagccatta	used for Fig. 2C
REALpgn1674R1	atttcacgcttacccaaacg	used for Fig. 2C
REALpgn1673F2	tcgctcgtgaacgagtaatg	used for Fig. 2C
REALpgn1673R2	gaatcgggcgtaggacagta	used for Fig. 2C
REALpgn0022F1	catggagaggaaatccctga	used for Fig. 2C
REALpgn0022R1	catttgcatcgggaaagtct	used for Fig. 2C
REALpgn0023F1	ctctgtgccatcgctgaata	used for Fig. 2C, Supplemental Fig. S10A
REALpgn0023R1	cttggacagccagggtgtat	used for Fig. 2C, Supplemental Fig. S10A
REALpgn1534F1	aggattccatctgccctctt	used for Fig. 2C
REALpgn1534R1	gaacgctcgcctttgactac	used for Fig. 2C
REALpgn1351F1	ccgcagaaaatcagaccatt	used for Fig. 2C
REALpgn1351R1	ctccgctcttccatcgatac	used for Fig. 2C
REALpgn0123F1	ttgctttgtgctttggtcag	used for Fig. 2C, 2E
REALpgn0123R1	cccagcccgatattagtcaa	used for Fig. 2C, 2E
REALpgn1676F3	agctcaatccggatcaaatg	used for Supplemental Fig. S10A
REALpgn1676R3	gatgatattgccgctttcgt	used for Supplemental Fig.S10A
REALpgn1675F2	aaacccgcaatggaatatca	used for Supplemental Fig. S10A
REALpgn1675R2	tctgctcttccggcagtatt	used for Supplemental Fig. S10A

REALpgn1674F3	ctcctgaccaagctccaaag	used for Supplemental Fig. S10A
REALpgn1674R3	catgttctcgggagaaagga	used for Supplemental Fig. S10A
REALpgn0778F2	ttgtacccgaagggagtacg	used for Supplemental Fig. S10A
REALpgn0778R2	tggatcgaacggagaaagag	used for Supplemental Fig. S10A
REALpgn0832F1	cgcaagaactaagcggaatc	used for Supplemental Fig. S10A
REALpgn0832R1	ctgataaacctgccccgttgt	used for Supplemental Fig. S10A
REALpgn2001F1	aattgaggatgccgaatgag	used for Supplemental Fig. S4B
REALpgn2001R1	ctgcatacgagcctttctcc	used for Supplemental Fig. S4B
pg16SrF1	gttgacttcagtggcggca	used for Fig. 2C, 2E, Supplemental Fig. S4B, S10A
pg16SrR1	agggaagacggttttcacca	used for Fig. 2C, 2E, Supplemental Fig. S4B, S10A
Halo-CTD fusion		
kgp-U400-KpnI	ggtaccactttaagtgttgttagacaacac	KpnI-kgp-U
kgp-sigR-SalI	gtcgacggcgtaaagaccaactcccaaaag	SalI-Kgp signal seq 19 aa
halo-F-SalI	gtcgacatggcagaaatcggtactggctt	SalI-HaloTag ORF-F
halo-stopR-BglII	agatctttaaccggaaatctccagagtaga	BglII-HaloTag ORF-stop-R
halo-R-BglII	agatctaccggaaatctccagagtagaaag	BglII-HaloTag ORF-R
rgpB-CTD73-F-BglII	agatcttctattgccgacgtagccaatgat	BglII-RgpB CTD (1990-2013)
rgpB-CTD-R-NotI	gcggccgcttacttcactataaccttttc	NotI-RgpB CTD (2211-2190)
porA-CTD84-F-BglII	agatctgatttgactaatatcgggctggggcgt	BglII-PorA CTD (487-510)
porA-CTD-R-NotI	gcggccgcttacttaatcagatacttctga	NotI-PorA CTD (741-720)
ppad-CTD84-F-BglII	agatctggagctgccaaagctcttcgtgcatgg	BglII-Pad CTD (1417-1440)
ppad-CTD-R-NotI	gcggccgcttatttgagaattttcattgtc	NotI-Pad CTD (1671-1650)
kgp-CTD72-F-BglII	agatctggagtggcagacgtaactgctcagaag	BglII-Kgp CTD (4954-4977)
kgp-CTD-R-NotI	gcggccgcttacttgatagcgagtttctct	NotI-Kgp CTD (5172-5151)
hbp35-CTD67-F-BglII	agatctgctacagaacaaattgttgctacc	BglII-Hbp35 CTD (832-855)
hbp35-CTD-R-NotI	gcggccgctcaaggaactaagactttaagg	NotI-Hbp35 CTD (1035-1012)
mfa5-CTD84-F-BglII	agatcttatgacgaagagtgggttgaatcggca	BglII-Mfa5 CTD (3433-3456)
mfa5-CTD-R-NotI	gcgccgcttagttgactacaactttcctt	NotI-Mfa5 CTD (3687-3665)

Supplemental Table S3. X-ray data collection and refinement statistics.

Crystal	Native	SeMet-derivative
Space group	*C*2	*C*2
Cell dimensions		
a, b, c (Å)	67.3, 43.2, 48.6	67.6, 43.3, 48.8
α, β, γ (°)	90.0, 108.5, 90.0	90.0, 108.7, 90.0
Wavelength	1.00	0.979
Resolution	46.1-1.3 (1.32-1.3)	35.9-1.35 (1.37-1.35)
R_{merge}	0.038 (0.329)	0.049 (0.648)
I/sI	17.9 (3.9)	13.4 (2.1)
Completeness (%)	95.5 (95.6)	96.9 (96.6)
Redundancy	3.8 (3.9)	4.8 (4.8)

Resolution range (Å)	46.1-1.3	(1.34-1.30)
No. of reflections working	29,538	(2,683)
No. of reflections test	1,590	(145)
R_w (%)	18.1	(22.6)

R_{free} (%)	20.3	(25.9)
Rms deviation bond length (Å)	0.009	
Rms deviation Bond angle (°)	1.057	
B-factors		
Protein atoms	18.8	
Solvent atoms	29.3	
Ramachandran plot (%)		
Most favored	99.2	
Allowed	0.8	
Disallowed	0	
No. of protein atoms	1, 004	
No. of solvent atoms	145	

Values in parentheses are for the highest resolution shell.

$R_w = \sum \| F_o | - | F_c \| / \sum | F_o |$, $R_{free} = \sum \| F_o | - | F_c \| / \sum | F_o |$

Fig. S1

A

WT *PGN_1770*

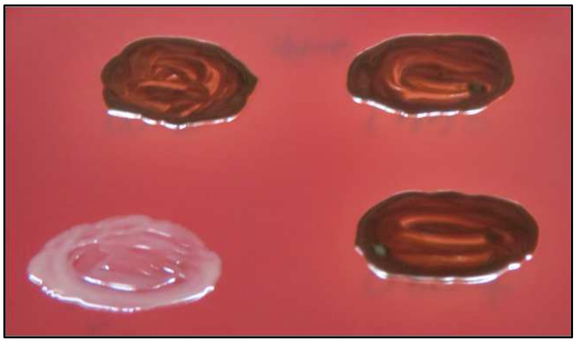

porA *PGN_0654*

B

WT *porA porY* *porA porY /porY(S266W)*

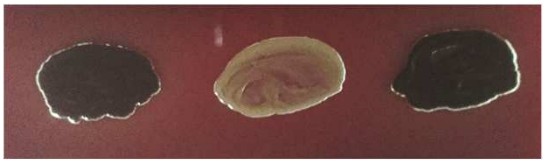

Fig. S2

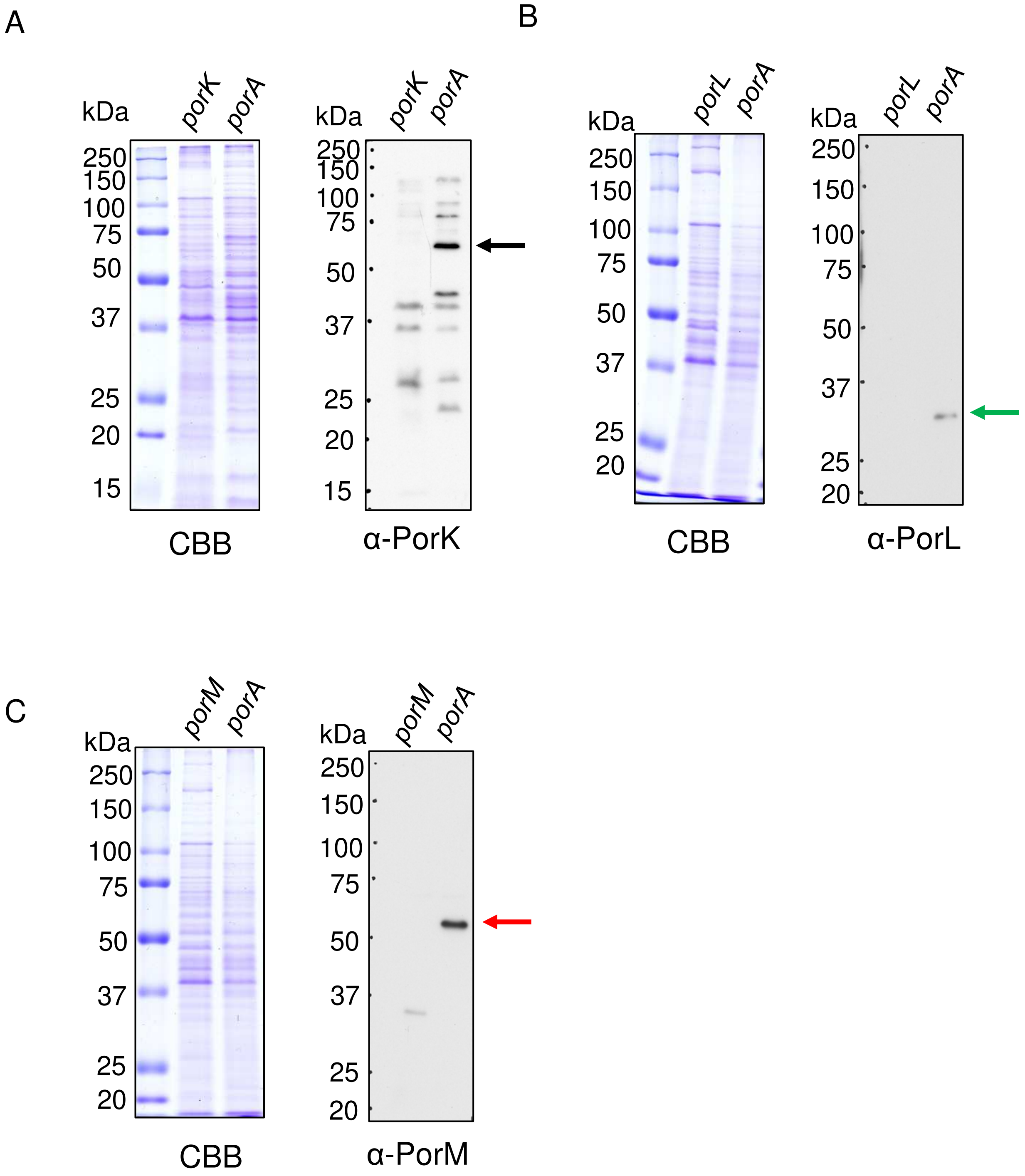

A
B
C
kDa
porK
porA
porL
porM
250
150
100
75
50
37
25
20
15
CBB
α-PorK
α-PorL
α-PorM

Fig. S3

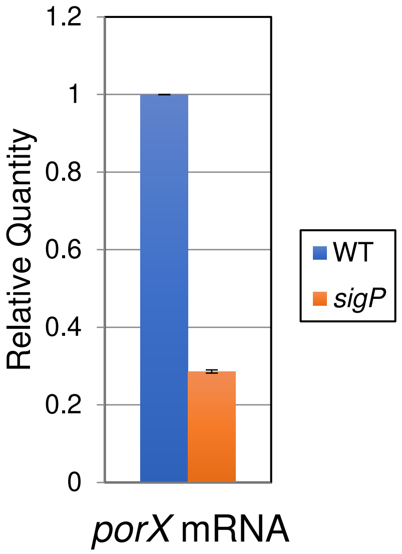

Fig. S4

A

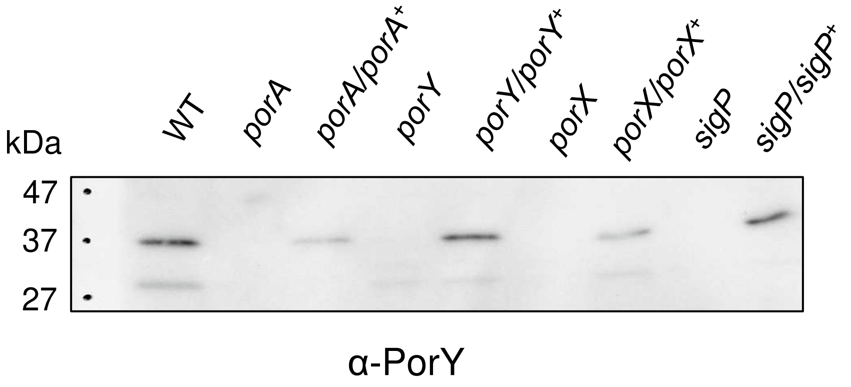
WT
porA
porA/porA+
porY
porY/porY+
porX
porX/porX+
sigP
sigP/sigP+
kDa
47
37
27
α-PorY

B

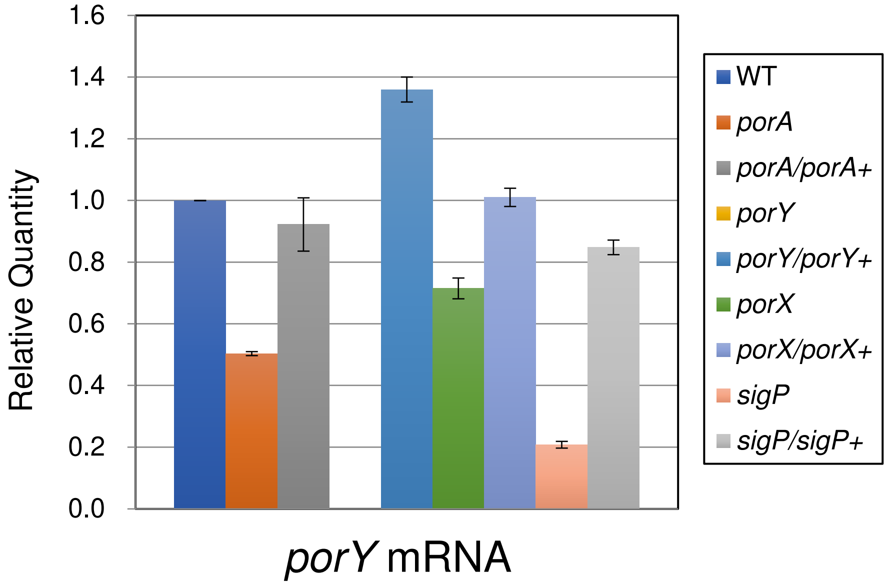
1.6
1.4
1.2
1.0
0.8
0.6
0.4
0.2
0.0
Relative Quantity
porY mRNA
WT
porA
porA/porA+
porY
porY/porY+
porX
porX/porX+
sigP
sigP/sigP+

Fig. S5

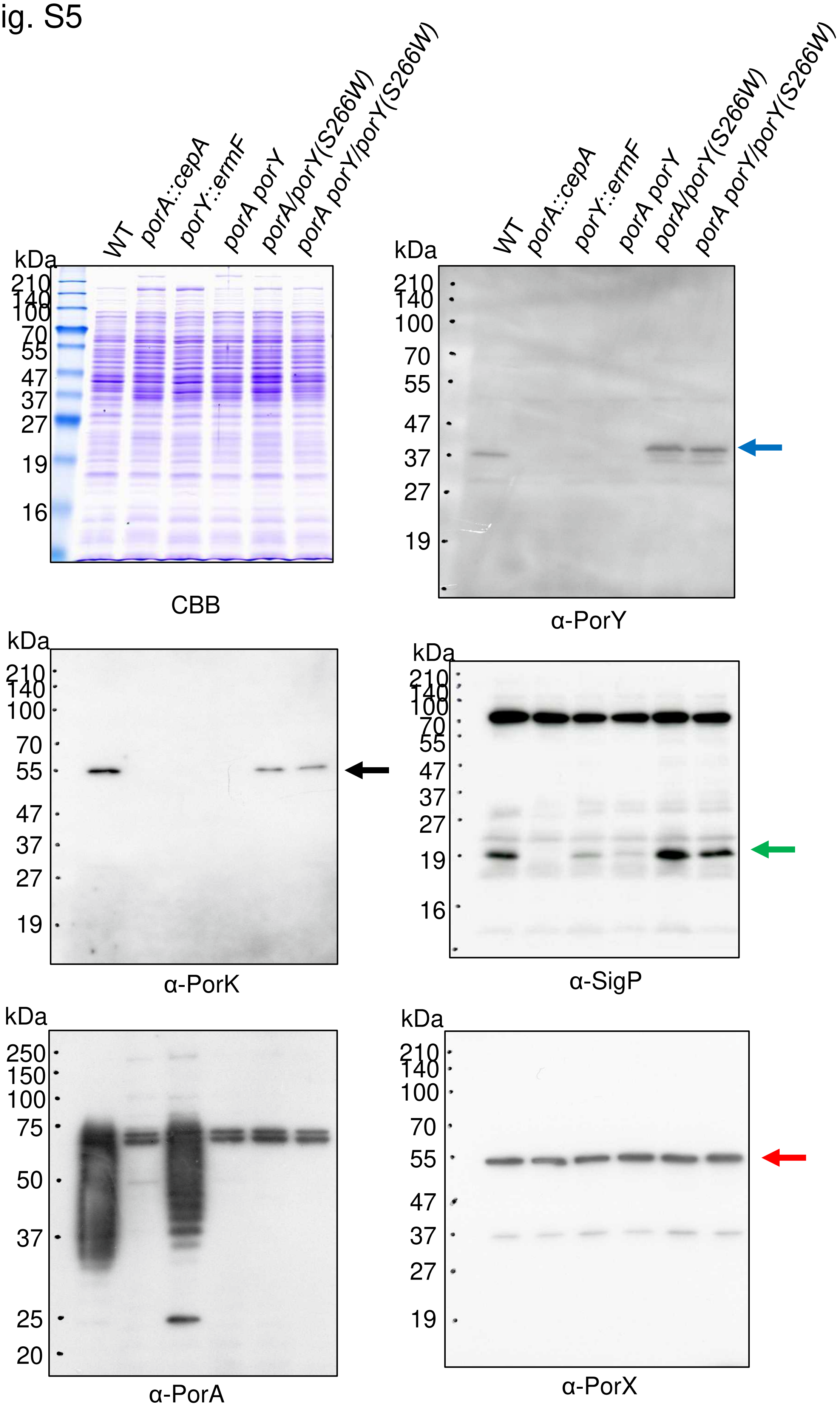
WT
porA::cepA
porY::ermF
porA porY
porA/porY(S266W)
porA porY/porY(S266W)
kDa
210
140
100
70
55
47
37
27
19
16
CBB
α-PorY
α-PorK
α-SigP
250
150
100
75
50
37
25
20
α-PorA
α-PorX

Fig. S6

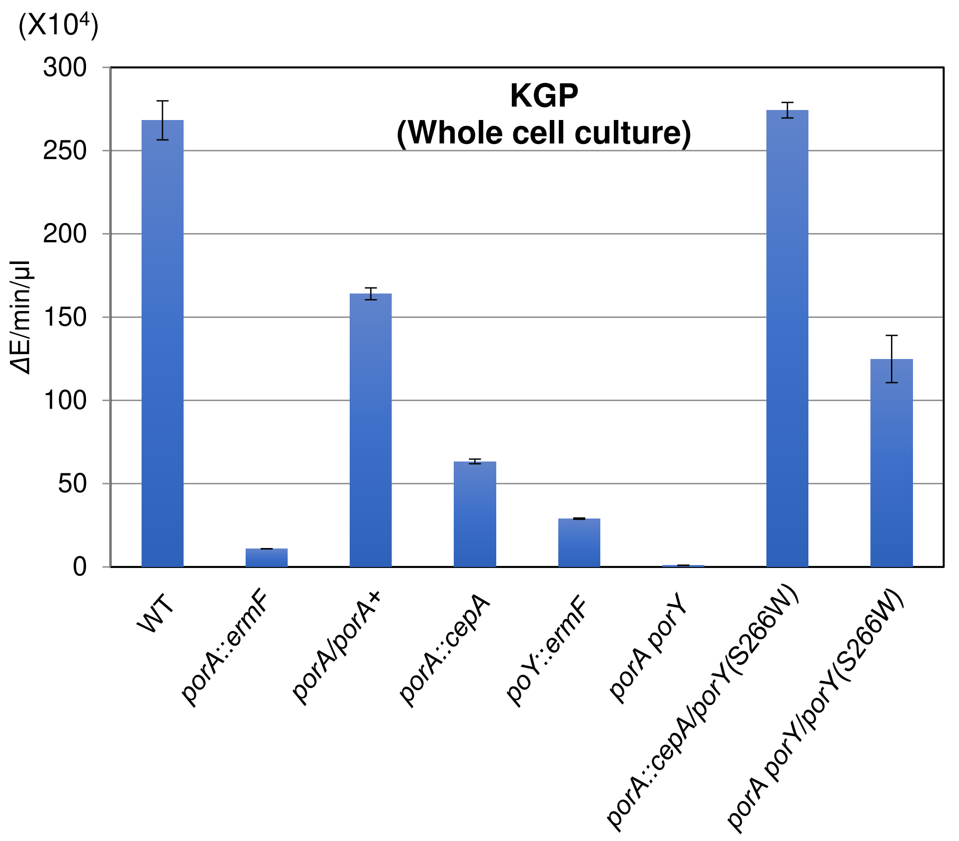

(X10⁴)
KGP
(Whole cell culture)
ΔE/min/μl
300
250
200
150
100
50
0
WT
porA::ermF
porA/porA+
porA::cepA
poY::ermF
porA porY
porA::cepA/porY(S266W)
porA porY/porY(S266W)

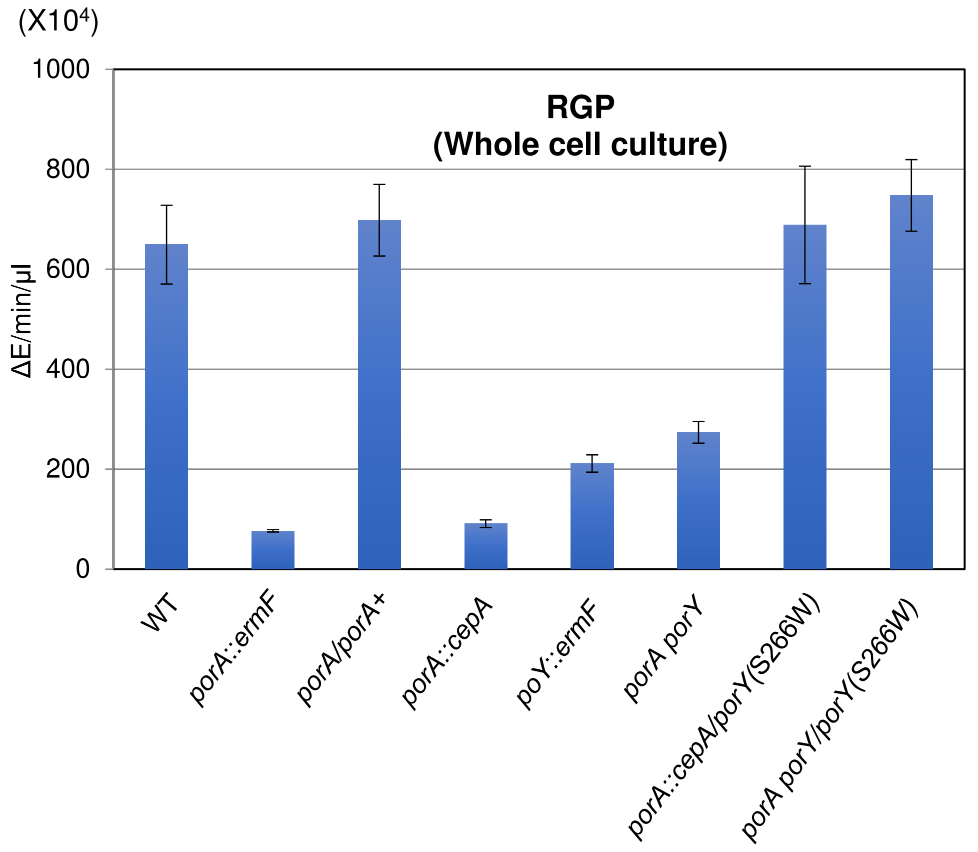

(X10⁴)
RGP
(Whole cell culture)
ΔE/min/μl
1000
800
600
400
200
0
WT
porA::ermF
porA/porA+
porA::cepA
poY::ermF
porA porY
porA::cepA/porY(S266W)
porA porY/porY(S266W)

Fig. S7

A

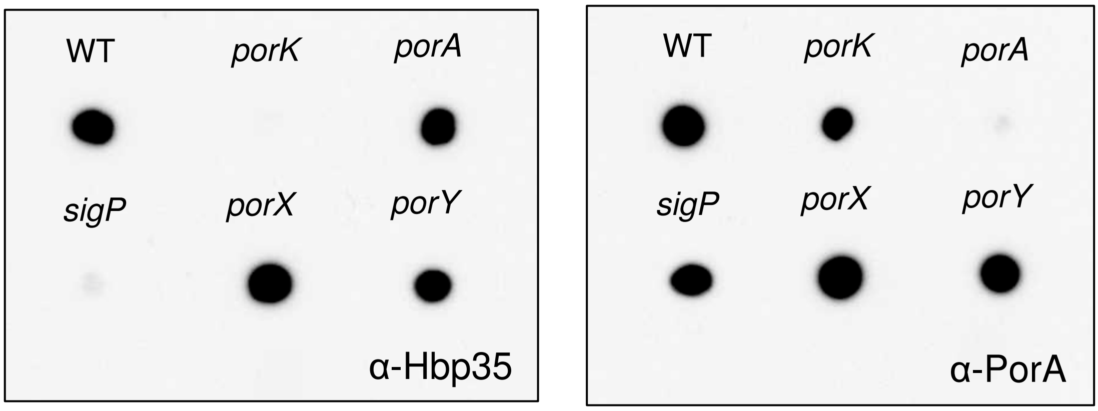
WT
porK
porA
sigP
porX
porY
α-Hbp35
WT
porK
porA
sigP
porX
porY
α-PorA

B

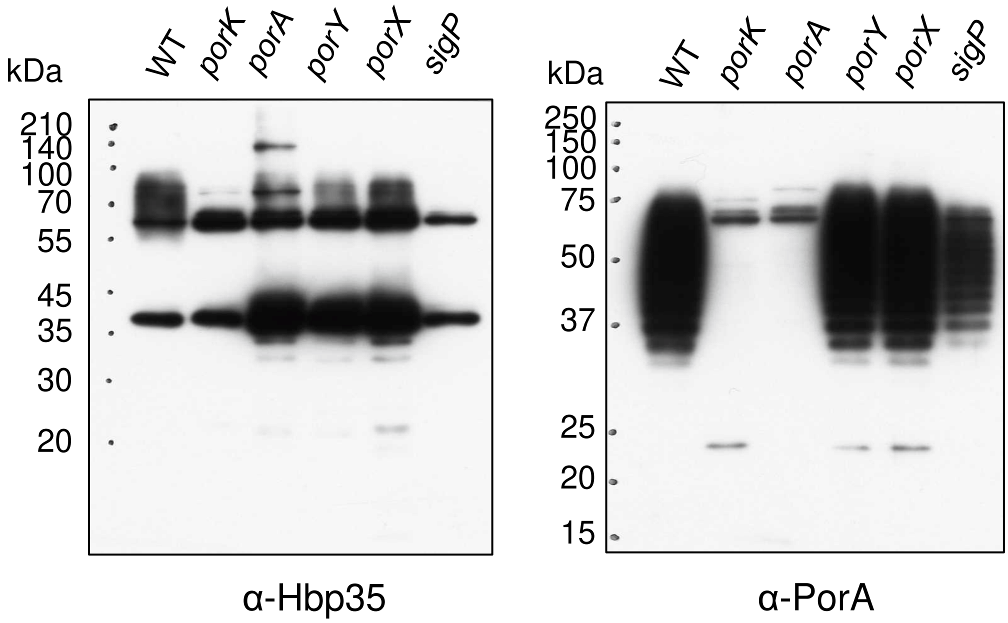
kDa
WT
porK
porA
porY
porX
sigP
210
140
100
70
55
45
35
30
20
α-Hbp35
kDa
WT
porK
porA
porY
porX
sigP
250
150
100
75
50
37
25
20
15
α-PorA

Fig. S8

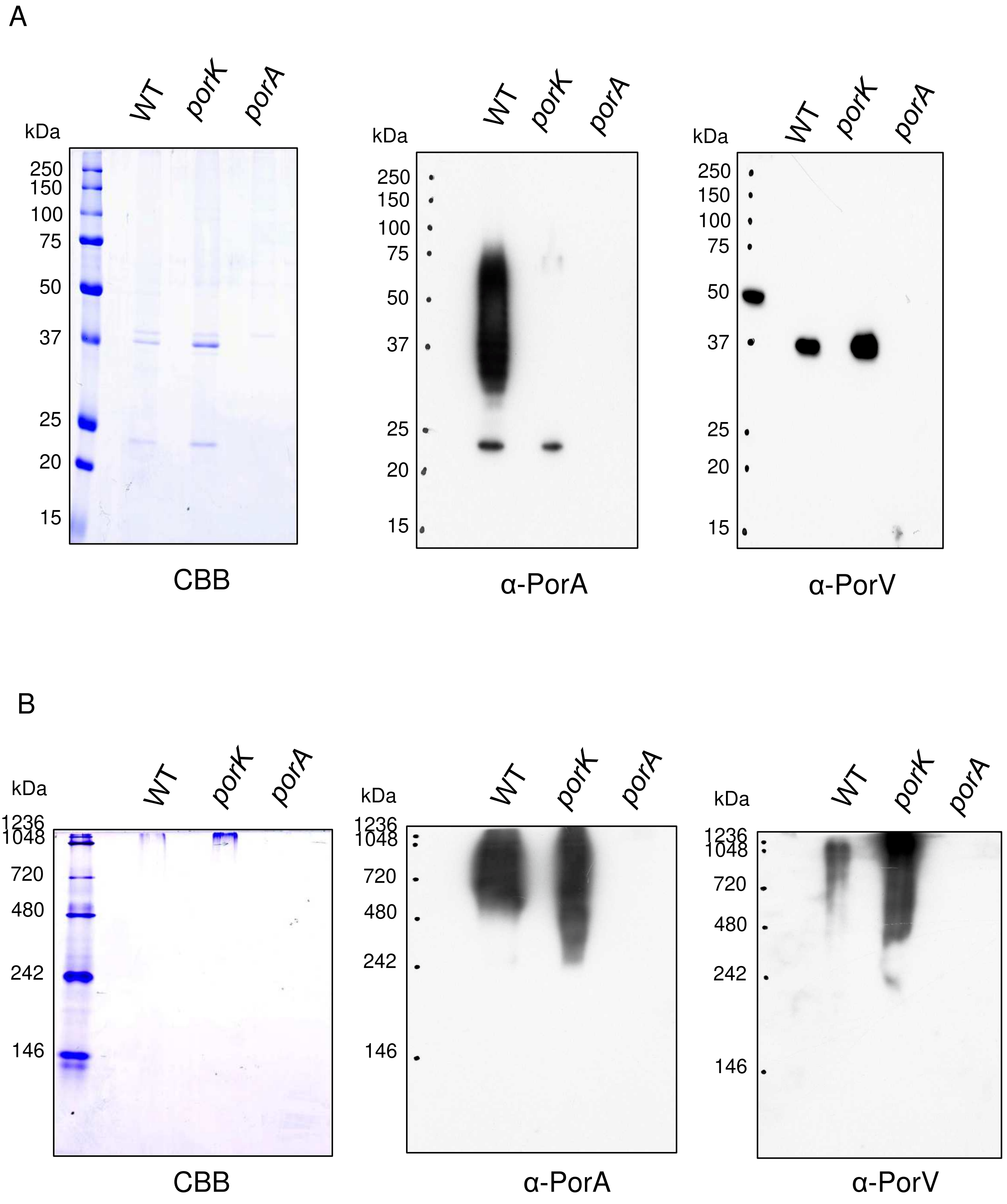
A
WT
porK
porA
kDa
250
150
100
75
50
37
25
20
15
CBB
α-PorA
α-PorV
B
WT
porK
porA
kDa
1236
1048
720
480
242
146
CBB
α-PorA
α-PorV

Fig. S9

A

```
RgpB   SIADVANDKPYTVAVS--GKTITVES----PAAGLTIFDMNGRRVATAK-------NRMVFEAQ---NGVYAVRIATEGK-TYTEKVIVK
Kgp    GVADVTAQKPYTLTVV--GKTITVTC----QGEAM-IYDMNGRRLAAGR-------NTVVYTAQ---GGYYAVMVVVDGK-SYVEKLAIK
Hbp35  ATEQIVATPSVKAYVQ--NGKIVVEE----EYSKMEVFNATG-QLVKNE-------SLVP--------GVYVVRITANGV-MHFLKVLVP
PorA   DLTNIGLGR-IALIQS--GNTCTLQYNSNGKRLALEVYNLLGVKVFTSQLPAGSGSYTLPVRLQ---RGVHIFRITEGGKPAFVQKYLIK
PPAD   -GAAKALRAWFNAGRS--ELAVSVSLN-IAGTYRIKLYNTAGEEVAAMTKELVAGTSVFSMDVYSQAPGTYVLVVEGNGI-RETMKILK
Mfa5   YDEEWVESAEVSVLVGTVGKRILITNN-CEHACQANVYTTDGKLLIRLDVK--PGSKSMTEPLI---DGAYVVSLQSPATSSNVRKVVVN
                              :            ::   *  :             .         * : . :   .      *
```

B

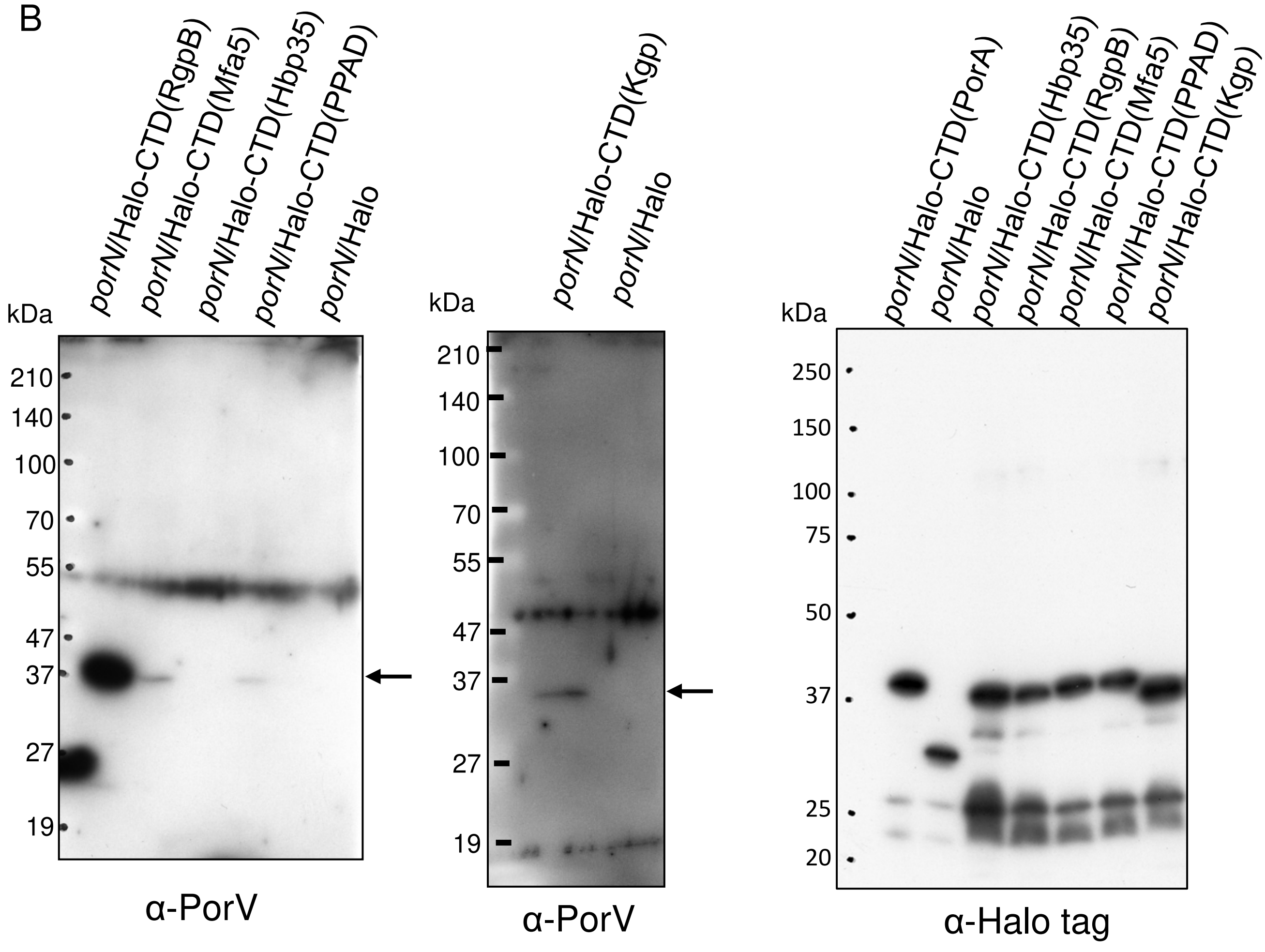

Fig. S10

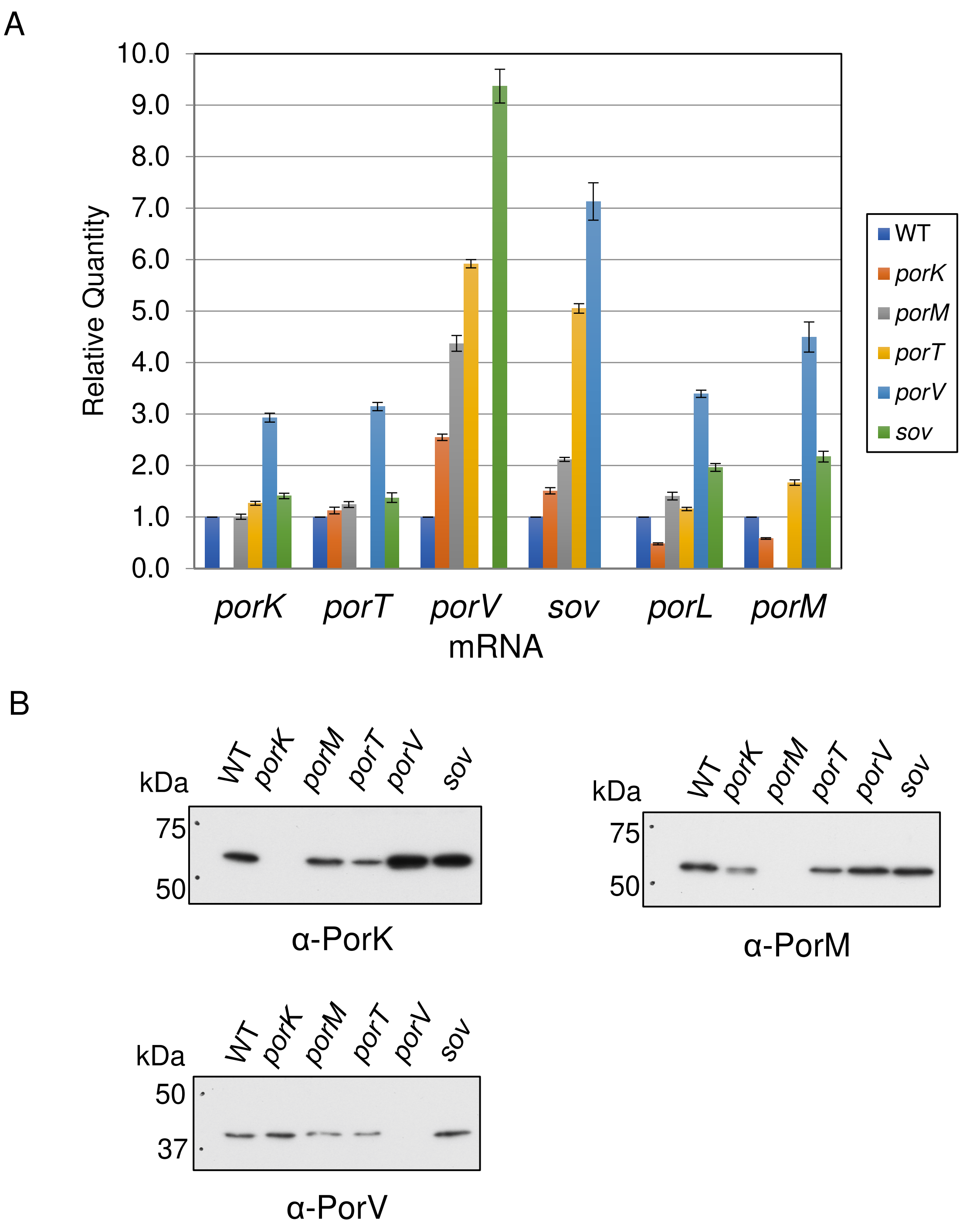
A
10.0
9.0
8.0
7.0
6.0
5.0
4.0
3.0
2.0
1.0
0.0
Relative Quantity
porK
porT
porV
sov
porL
porM
mRNA
WT
porK
porM
porT
porV
sov
B
kDa
WT
porK
porM
porT
porV
sov
75
50
α-PorK
kDa
WT
porK
porM
porT
porV
sov
75
50
α-PorM
kDa
WT
porK
porM
porT
porV
sov
50
37
α-PorV

Fig. S11

E

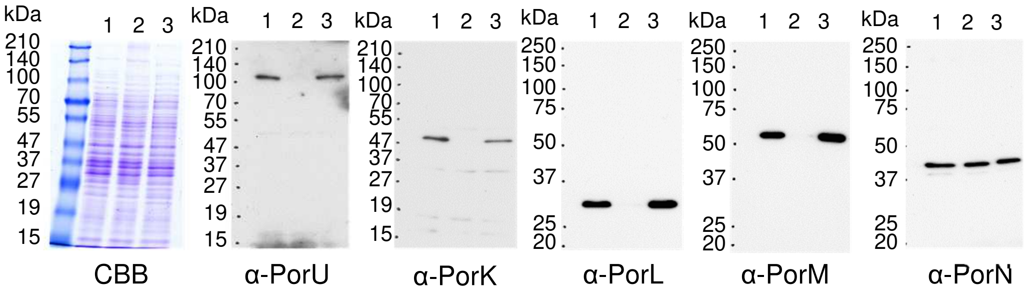
kDa
1 2 3
210
140
100
70
55
47
37
27
19
15
CBB
α-PorU
α-PorK
α-PorL
α-PorM
α-PorN
250
150
75
50
25
20

F

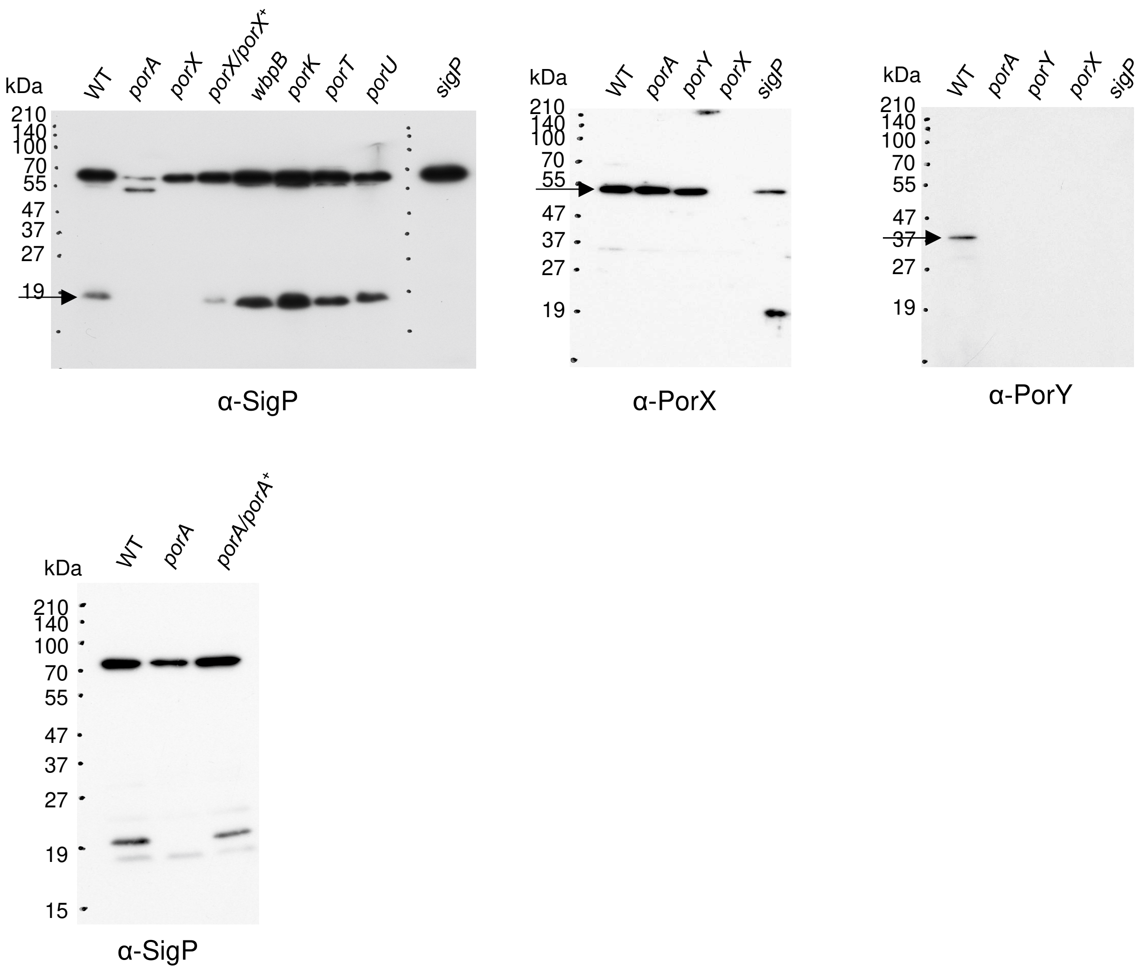
kDa
WT
porA
porX
porX/porX⁺
wbpB
porK
porT
porU
sigP
porY
porA/porA⁺
210
140
100
70
55
47
37
27
19
15
α-SigP
α-PorX
α-PorY
α-SigP

Fig. S12

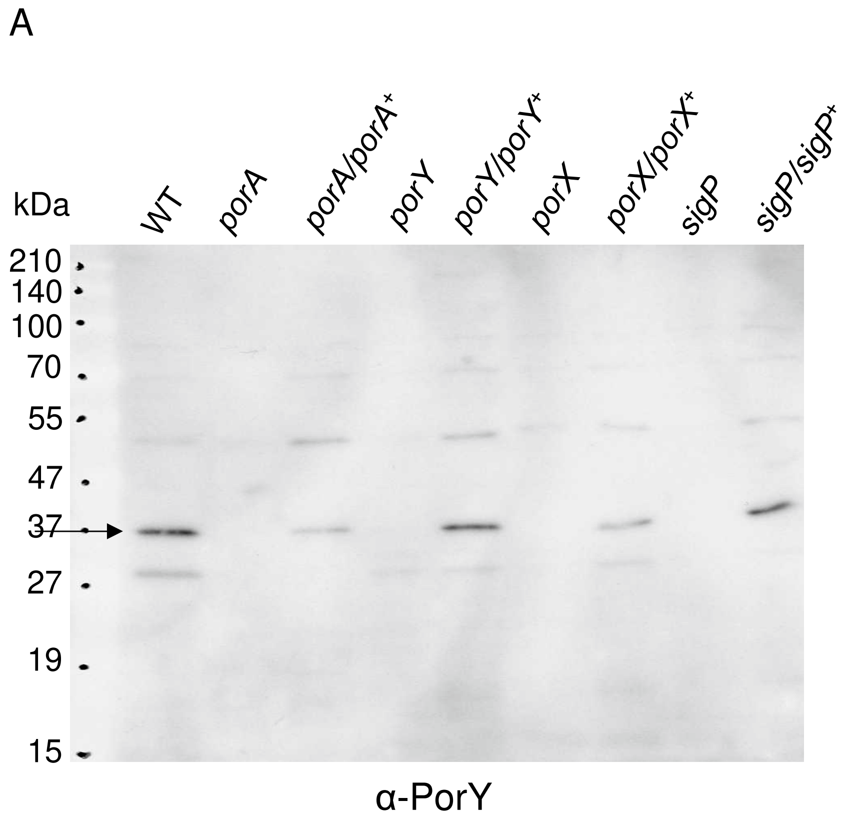
A
kDa
WT
porA
porA/porA+
porY
porY/porY+
porX
porX/porX+
sigP
sigP/sigP+
210
140
100
70
55
47
37
27
19
15
α-PorY

Fig. S13

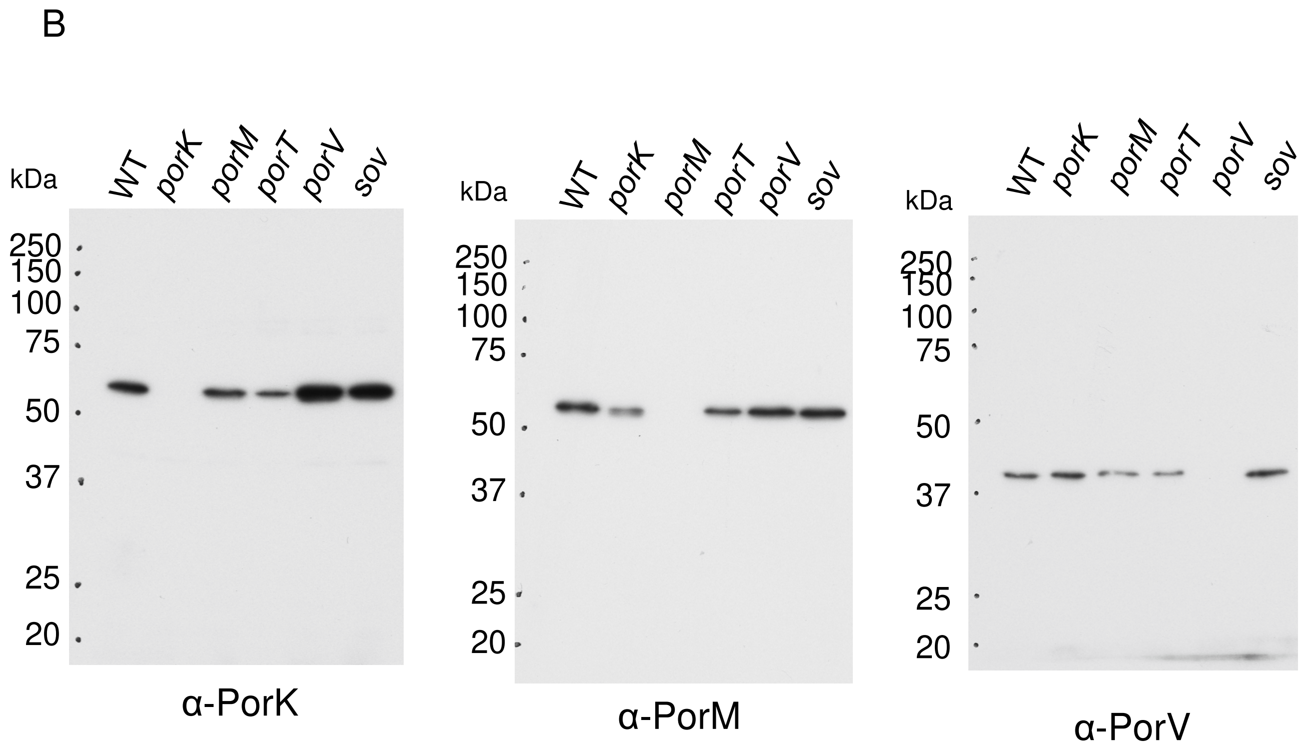
B
kDa
WT
porK
porM
porT
porV
sov
250
150
100
75
50
37
25
20
α-PorK
α-PorM
α-PorV
